# Computational Approaches in Preclinical Studies on Drug Discovery and Development

**DOI:** 10.3389/fchem.2020.00726

**Published:** 2020-09-11

**Authors:** Fengxu Wu, Yuquan Zhou, Langhui Li, Xianhuan Shen, Ganying Chen, Xiaoqing Wang, Xianyang Liang, Mengyuan Tan, Zunnan Huang

**Affiliations:** ^1^Key Laboratory of Big Data Mining and Precision Drug Design of Guangdong Medical University, Research Platform Service Management Center, Dongguan, China; ^2^Key Laboratory of Pesticide & Chemical Biology, Ministry of Education, College of Chemistry, Central China Normal University, Wuhan, China; ^3^The Second School of Clinical Medicine, Guangdong Medical University, Dongguan, China; ^4^Key Laboratory for Research and Development of Natural Drugs of Guangdong Province, School of Pharmacy, Guangdong Medical University, Dongguan, China; ^5^Marine Biomedical Research Institute of Guangdong Zhanjiang, Zhanjiang, China

**Keywords:** drug discovery, pre-clinical studies, ADMET, pharmacokinetics, PBPK modeling

## Abstract

Because undesirable pharmacokinetics and toxicity are significant reasons for the failure of drug development in the costly late stage, it has been widely recognized that drug ADMET properties should be considered as early as possible to reduce failure rates in the clinical phase of drug discovery. Concurrently, drug recalls have become increasingly common in recent years, prompting pharmaceutical companies to increase attention toward the safety evaluation of preclinical drugs. *In vitro* and *in vivo* drug evaluation techniques are currently more mature in preclinical applications, but these technologies are costly. In recent years, with the rapid development of computer science, *in silico* technology has been widely used to evaluate the relevant properties of drugs in the preclinical stage and has produced many software programs and *in silico* models, further promoting the study of ADMET *in vitro*. In this review, we first introduce the two ADMET prediction categories (molecular modeling and data modeling). Then, we perform a systematic classification and description of the databases and software commonly used for ADMET prediction. We focus on some widely studied ADMT properties as well as PBPK simulation, and we list some applications that are related to the prediction categories and web tools. Finally, we discuss challenges and limitations in the preclinical area and propose some suggestions and prospects for the future.

## Introduction

**Figure 1:**
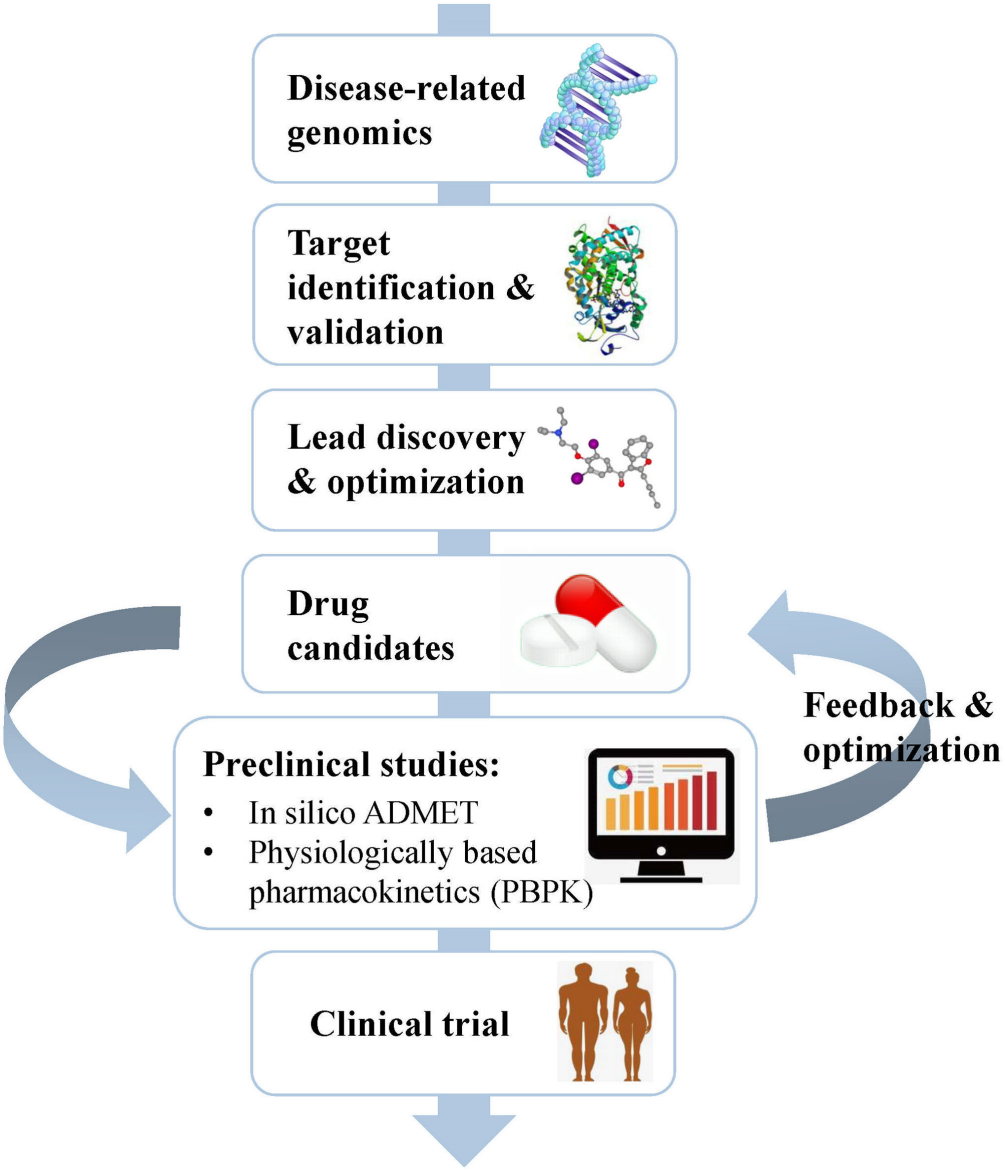
Schematic flow chart summarizing the process of drug discovery and the main content of the preclinical study. Preclinical studies mainly include *in silico* ADMET prediction and PBPK simulation, which play important roles in helping the selection and optimization of drug candidates.

Drug development is a complicated, risky, and time-consuming process that can be divided into several stages, including disease-related genomics, target identification and validation, lead discovery and optimization, preclinical studies, and clinical trials (Tang et al., 2006) (Figure 1). During early drug discovery, the activities and specificities of candidate drugs are usually assessed at an early stage, and pharmacokinetics and toxicities are evaluated at a relatively late stage (Selick et al., 2002). However, the undesirable efficacy and safety, mainly caused by absorption, distribution, metabolism, excretion, and toxicity (ADMET) characteristics, resulted in the failure of many candidate drugs in the final stage (Caldwell et al., 2009). Cook et al. (2014) comprehensively reviewed the results of AstraZeneca's small-molecule drug projects from 2005 to 2010 based on a longitudinal study. They found that unacceptable safety and toxicity were the most important reasons for the failure of more than half of all project closures. As with the development of drug discovery, it was realized that it is important to filter and optimize the ADMET properties for drugs at an early stage, which has been accepted and widely used to reduce the attrition rate in drug research and development. A “fail early, fail cheap” strategy has been employed by many pharmaceutical companies (Yu and Adedoyin, 2003). Pharmacokinetics and toxicity assessments of preclinical drugs are of great value in reducing the failure rate of new chemical entities (NCEs) in clinical trials (Kola and Landis, 2004; Yang et al., 2018b; Ferreira and Andricopulo, 2019). In recent years, *in vitro* and *in vivo* ADMET prediction methods have been widely used, but it is impractical to perform complex and expensive ADMET experiments on a large number of compounds (Cheng et al., 2013; Patel C. N. et al., 2020). Thus, an *in silico* strategy to predict ADMET properties has become very attractive as a cost-saving and high-throughput alternative to experimental measurement methods.

**Figure 2:**
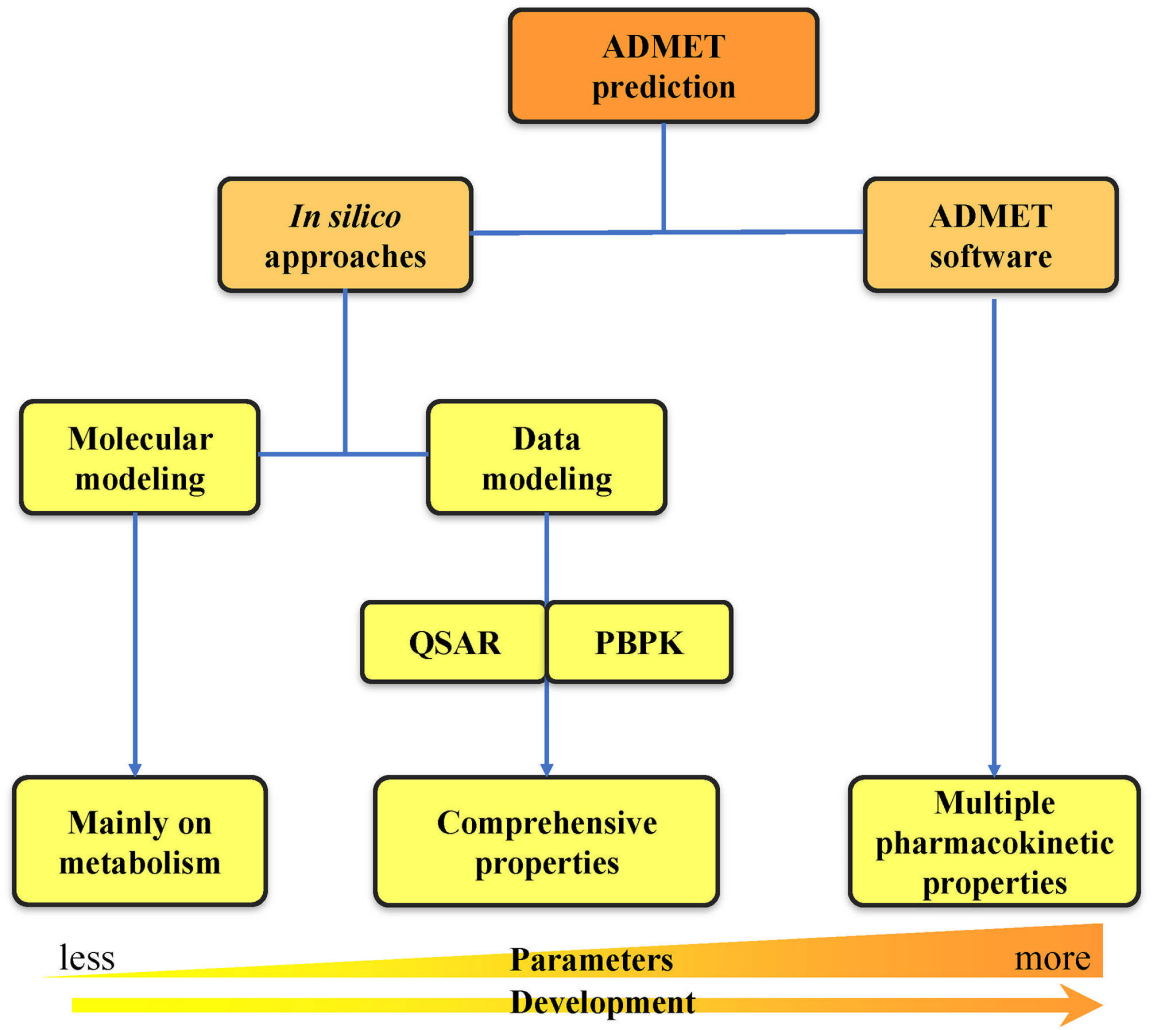
Classification of ADMET prediction strategies. The ADMET prediction includes the primary *in silico* approaches and the usage of ADMET software. The development from *in silico* approaches to ADMET software has undergone a long process of predicting property parameters from less to more.

With the rapid development of computer technologies, the high-throughput screening of compounds, application of combinatorial chemistry, and ability of compound synthesis have increased dramatically. The early demands for ADMET data on lead compounds have also significantly increased, and methods for evaluating ADMET *in vitro* are gradually increasing. Many *in silico* methods have been successfully applied to the *in vitro* prediction of ADMET, and *in silico* models have also been developed to replace *in vivo* models for the prediction of pharmacokinetics, toxicity, and other parameters (Zhu et al., 2011; Wang et al., 2015; Alqahtani, 2017). *In silico* ADMET prediction has progressed with the continuous development of cheminformatics and has entered the era of big data (Ferreira and Andricopulo, 2019). Two *in silico* approach categories can be used for ADMET prediction: molecular modeling and data modeling. Molecular modeling is based on the three-dimensional structures of proteins. It includes multiple methods such as molecular docking, molecular dynamics (MD) simulation, and quantum mechanics (QM) calculation (Bowen and Guener, 2013; Cheng et al., 2013; Silva-Junior et al., 2017). Data modeling includes quantitative structure–activity relationship (QSAR) (Cumming et al., 2013) and physiologically-based pharmacokinetic (PBPK) modeling (Fan and de Lannoy, 2014). Due to the increase in number of properties that need to be predicted, a series of ADMET software programs capable of comprehensive property prediction have been developed. The development from *in silico* approaches to ADMET software has undergone a long process of predicting property parameters from less to more at early to late timepoints (Figure 2). This review first provides a detailed introduction to the two *in silico* approaches of ADMET prediction. Then, we summarize the widely used databases and software related to ADMET prediction. Finally, we analyze the problems and challenges faced by computer model prediction methods as well as the tools, and we propose some of our own prospects for future development in this area.

## *In silico* Approaches

### Molecular Modeling

**Figure 3:**
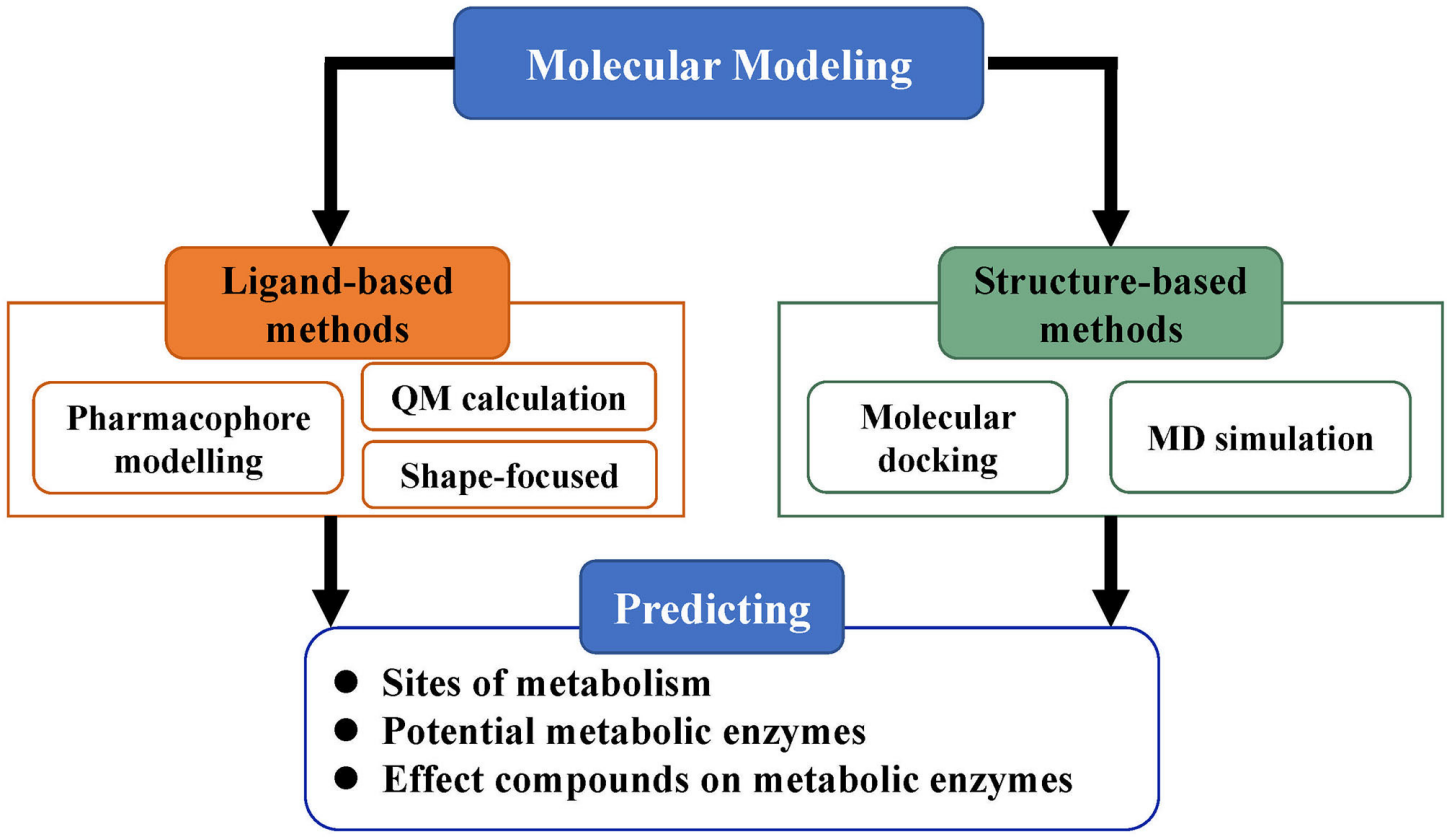
Strategy of molecular modeling in ADMET prediction. Molecular modeling is divided into ligand-based methods and structure-based methods and mainly used for the prediction of metabolic sites, potential metabolic enzymes, and effects of compounds on metabolic enzymes.

Molecular modeling, based on the three-dimensional structures of proteins, is an important category in predicting ADMET properties and includes methods such as pharmacophore modeling, molecular docking, MD simulations, and QM calculations (Figure 3). As more and more three-dimensional structures of ADMET proteins become available, molecular modeling can complement or even surpass QSAR studies (Moroy et al., 2012). Applying molecular modeling to perform ADMET prediction is a challenge because the ADMET proteins usually have flexible and large binding cavities. Many promising results of molecular modeling in predicting compound metabolism have been reported. The methods in these cases can be generally divided into ligand-based and structure-based and help not only to analyze metabolic properties but also to further optimize compound toxicity, bioavailability, and other parameters (Lin et al., 2003).

#### Ligand-Based Methods

Ligand-based methods derive information on proteins' active sites based on the shapes, electronic properties, and conformations of inhibitors, substrates or metabolites; this information depends on the assumption that the metabolic properties of compounds are entirely the result of their chemical structures and characteristics (de Groot et al., 2004; Andrade et al., 2014). In this category, pharmacophore modeling is one of the most widely used methods. The interactions between ligands and receptors can be predicted by constructing a pharmacophore model to cover the structures or properties of ligands in three-dimensional space and then to simulate the spatial and chemical properties of binding sites (de Groot, 2006). Therefore, the availability of ligand data is essential to the construction of pharmacophore models. In recent years, there have been many cases of using pharmacophore models to screen promising compounds with outstanding ADMET properties (Nandekar et al., 2016; El-Zahabi et al., 2019; Mohan et al., 2020; Patel D. B. et al., 2020; Rawat and Verma, 2020). For example, Nandekar et al. (2016) generated and validated a pharmacophore model to screen anticancer compounds acting via cytochrome P450 1A1 (CYP1A1). Nine compounds that have preferred pharmacophore characteristics and are capable of generating reactive metabolites were finally selected for further study. Rawat and Verma (2020) developed a pharmacophore model to discover new dual target inhibitors of *Plasmodium falciparum* dihydroorotate dehydrogenase (PfDHODH) and cytochrome bc1 complex (PfCytbc1) to treat malaria. The molecule MMV007571, which has been validated as an efficient multi-target inhibitor, was used to extract features from the binding information for the model construction. The model was used to screen a library including more than 40,000 molecules. After a series of experiments, two compounds were developed with the desired properties in binding potential and pharmacokinetic characters.

The shape-focused method is another category of ligand-based methods. This method is based on the fact that the shapes of a ligand and the binding site of its receptor should be complementary. Thus, molecules that have a comparable shape should be able to bind to the same receptor. It is more possible for a ligand to bind with the same target if it has greater similarity with the reference molecule (Putta and Beroza, 2007). This method requires only one reference molecule (shape) to perform a screening. However, more models should be constructed to cover more diverse chemical space if different shapes are available (Perez-Nueno and Ritchie, 2011), particularly for highly flexible proteins. Some studies using the shape-focused method have been reported in recent years (Reddy et al., 2013; Chen et al., 2015; Kumar et al., 2015; Prabhu and Singh, 2019). For example, Chen et al. (2015) presented a shape-based virtual screening to find new cores for the design of acetylcholinesterase (AChE) inhibitors. The shape of commercial inhibitor tacrine was used to search for new potential inhibitors. Two hit compounds were finally identified with good ADMET properties and better activity than tacrine.

With the improvement of computer hardware performance, the time-consuming QM calculation in ADMET prediction has become possible and popular. The QM calculation can be used to evaluate the bond break, which is a step required for metabolic transformation (Andrade et al., 2014). Moreover, this calculation uses an accurate means of describing electrons in atoms and molecules (Modi, 2003). Hence, QM calculation is very helpful in ADMET prediction. Extensively increasing studies involving the application of QM methods have been conducted to describe ADMET properties of new compounds (Li et al., 2012; Taxak et al., 2013; Kavitha et al., 2015; Sasahara et al., 2015; Evangelista et al., 2016; Mondal et al., 2017). The *ab initio* (Hartree-Fock), semiempirical (AM1 and PM3), and density functional theory (DFT) approaches are most commonly used in these studies (Silva-Junior et al., 2017). For example, Mondal et al. (2017) used the DFT method to study the absorption profile and antimicrobial activity for five sulfonamide Schiff bases. The geometries compared well with the experimental value. Sasahara et al. (2015) used the DFT method to evaluate the metabolic selectivity of antipsychotic thioridazine by CYP450 2D6. This study revealed the importance of the substrate orientation in the reaction center of this enzyme for the metabolic reaction.

All ligand-based methods need to address the problem of the uncertainty of metabolic enzyme binding sites. If reliable structural data for a metabolic enzyme are lacking, the properties of ligand binding to the enzyme can only be speculative, and minor modifications to a ligand may cause a significant decrease in ligand-protein affinity (Kirchmair et al., 2012). Therefore, it is difficult to predict ADMET using ligand-based methods without reliable protein structure data. However, the use of ligand-based methods in metabolism prediction can easily eliminate inappropriate compounds and reduce the number of compounds that fail during the synthetic evaluation cycle and more expensive late stages.

#### Structure-Based Methods

Structure-based methods can be used not only to predict the ADMET properties of compounds, but also to study specific interactions between small molecules and ADMET proteins. In general, they focus on obtaining binding modes from the static structures of protein-ligand complexes, regardless of time-dependent conformational fluctuations (Cheng T. et al., 2012). For structure-based methods, changes in receptor conformation must be considered mainly because of the possible interaction between the existing large and flexible binding cavities with diverse ligands. By performing MD calculation to simulate the dynamic changes of spatial shape, we may obtain an adequate conformation sampling to search a stable and reliable binding mode for ADMET prediction. In recent years, structure-based methods have been widely used, for instance, to predict the binding patterns of substrates (Macalino et al., 2015), conformational changes in enzymes (Cheng T. et al., 2012), and their catalytic effects on physiological systems (Cui and Karplus, 2003), to evaluate substrate affinity, instability and metabolic pathways (Sun and Scott, 2010), and to assess the relationship between metabolism and carcinogenicity (Fratev and Benfenati, 2008).

The molecular modeling strategy makes important contributions to the rationalization of metabolic reactions of compounds, allowing the simulation of binding modes between drugs and macromolecules in the ADMET process at atomic or molecular levels. With the rapid development of structural elucidation and pharmacokinetic calculation techniques, structure-based methods are becoming increasingly predictive and accurate. However, the molecular modeling strategy is still limited by drawbacks such as a requirement to accurately analyze the structural flexibility of proteins (Kazmi et al., 2019). Additionally, high-resolution experimental structural data of the target will be more conducive to our accurate prediction of the drug metabolic fate (Doss et al., 2014). Surely, combining improved structure-based and ligand-based methods can create synergistic effects in metabolic prediction, enabling more comprehensive descriptions of metabolic reactions (Issa et al., 2017; Kar and Leszczynski, 2017).

### Data Modeling

There are two widely used data modeling methods to predict ADMET-related properties, QSAR, and PBPK modeling (Cheng et al., 2013). ADMET analysis and prediction in QSAR mainly depends on many molecular descriptors, including topological, geometrical physicochemical, or electronic descriptors. Many properties, such as blood-brain barrier (BBB), clinical adverse effects, percent protein binding (%PPB), lipophilicity (logP), preclinical toxicological endpoints, and metabolism of pharmaceutical substances, can be predicted using the QSAR method. The PBPK modeling always predicts parameters concerning the dose size and dose frequency, such as the volume of distribution at steady-state (Vss), total drug clearance (CL), and fraction of dose that reaches the portal vein (Fabs), because most drugs are taken orally.

#### QSAR

QSAR, which employs mathematical models to describe relationships between molecular structures and their biological activities, has been used in pharmaceutical chemistry since the 1960s (Hansch, 1981). The classical QSAR developed by Hansch was used to predict ADMET (Hansch, 1981). It is mainly based on the hypothesis that similar molecules exhibit similar properties (Patterson et al., 1996). Thus, two prediction methods are primarily considered: (1) prediction based on molecular similarity (pharmacophore- and molecular fragment-based methods) and (2) prediction based on property similarity (log P, log D, and others) (Yongye and Medina-Franco, 2013). In these two methods, the accuracy of prediction depends on the attribute characteristics in the applicability domain contained in the training set. Thus, when using a model constructed with a specific training set, prediction should be performed using compounds that have a similar structure space to those in the training set to improve the prediction accuracy of the QSAR model, since compounds with similar distribution in chemical space are more likely to exhibit similar biological activities (Huang and Fan, 2011).

**Figure 4:**
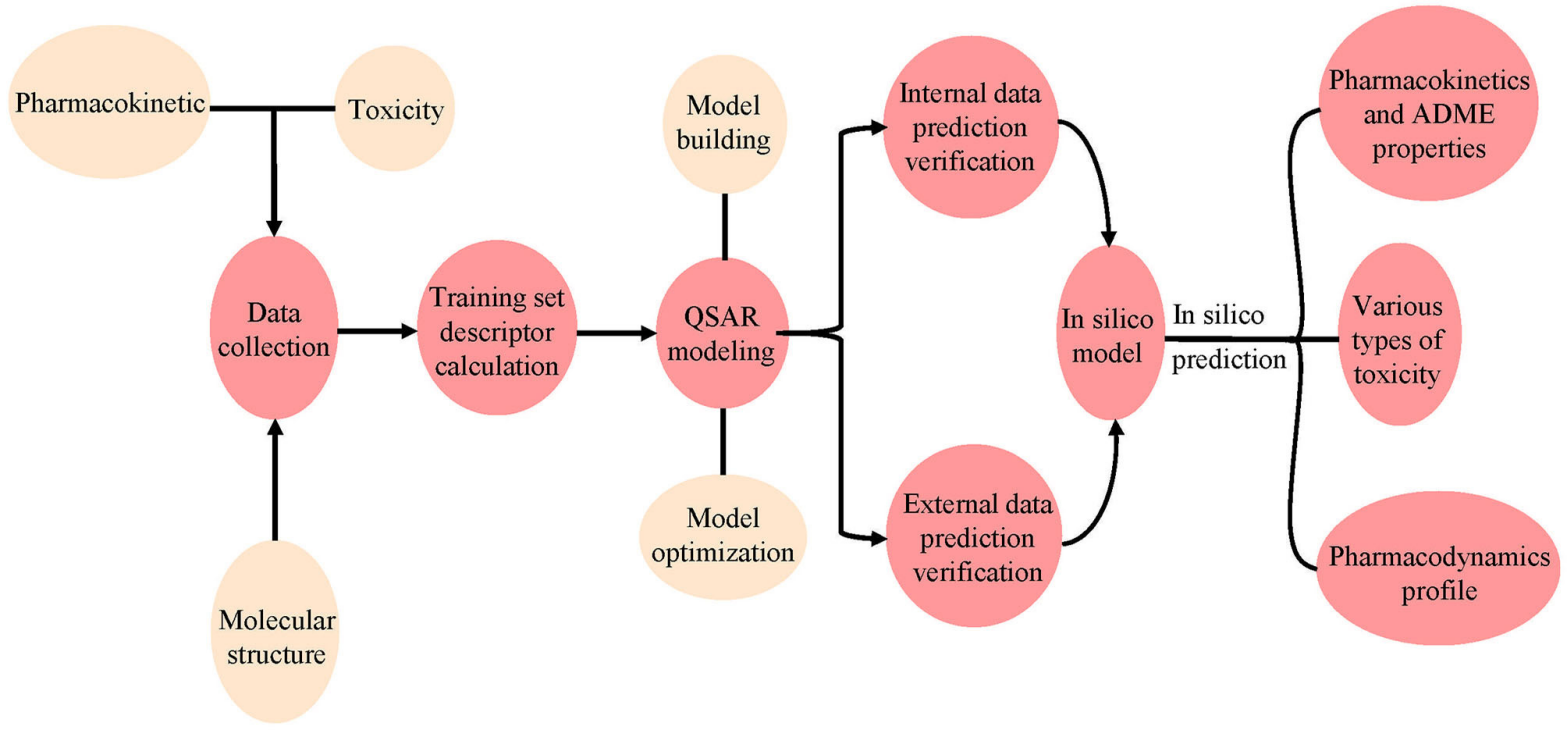
Workflow for the use of pharmacodynamic, pharmacokinetic, and toxicity databases and models. The molecular data were first collected from different databases, and the QSAR model was constructed using the collected or calculated molecular descriptors. Internal and external validation were then performed using the model. Finally, the validated model was used to predict ADMET properties for new chemicals.

QSAR is a method for using various biochemical and physical data to construct models. In QSAR studies, compounds can be mathematically codified as molecular descriptors, and the relationship between molecular descriptors and defined properties is constructed by statistical methods, after which a generated model is used to predict the corresponding properties of new compounds (Michielan and Moro, 2010). This method first transforms molecular structure into molecular descriptors, which are then used to establish prediction models by using statistical approaches or machine learning techniques such as support vector machine (SVM) and K Nearest Neighbor (kNN) (Wang S. et al., 2016; Wu et al., 2019; Yang et al., 2019; Fu et al., 2020). For example, Schyman et al. (2017) used the variable nearest neighbor (vNN) method to develop 15 ADMET prediction models and to use them to quickly assess some potential drug candidates, including toxicity, microsomal stability, mutagenicity, and likelihood of causing drug-induced liver injury. Belekar et al. (2015) developed a computational model to identify compounds as breast cancer resistance protein (BCRP) inhibitors or not by using various machine learning approaches like SVM, k-NN, and the artificial neural network (ANN). The prediction accuracy of all three approaches was over 85%. Finally, internal and external cross-validation were performed to confirm the reliability of the model before it is used on new predictions to find molecules outside the training set (Figure 4).

QSAR modeling uses a large number of descriptors that allow lookups, enable structure/response associations, and help with similarity and substructure searches (Khan, 2010). Most of the available descriptors can be divided into three categories: (1) two-dimensional molecular topology information; (2) three-dimensional molecular structure; and (3) physicochemical and electronic descriptors, which are commonly used to predict ADMET-related properties (Danishuddin and Khan, 2016; Tabeshpour et al., 2018; Zhang et al., 2018). Jiang et al. (2020) developed a series of QSAR models by using 379 molecular descriptors to discriminate BCRP inhibitors. The descriptors characterized the physicochemical, two-dimensional substructures, and drug-like properties of the studied compounds. Lapins et al. (2018) constructed a QSAR model to predict the lipophilicity of compounds by using a signature molecular descriptor, which is related to the molecular two-dimensional topology information from 1.6 million compounds. Xu et al. (2017) developed three deep learning-based QSAR models to evaluate the acute oral toxicity (AOT) of compounds. The atom and bond information extracted from over 2,000 two-dimensional molecule structures were used as descriptors to construct the models. The best model achieved an external prediction accuracy over 94%, which is more efficient than traditional fingerprints or descriptors. Bujak et al. (2015) predicted the permeability of the BBB of chemical compounds using molecular energy-related descriptors in combination with the well-known lipophilicity descriptors. The data indicate that the QSAR model has important information value, and these descriptors may have supportive value in predicting the blood brain distribution (Bujak et al., 2015). Therefore, accurate prediction of ADMET parameters mainly depends on the selection of a suitable modeling method, molecular descriptors of specific ADMET endpoints, and large experimental data sets related to these endpoints. Only in this way can the ADMET properties of the candidate compounds be predicted precisely.

At present, many tools used for ADMET prediction have been developed based on QSAR methods. These tools utilize different descriptors to define the collected data, and then the mathematical model fitted from the training set is used to predict the properties. We listed three widely used QSAR-based ADEMT prediction tools and related studies herein. (1) The Danish QSAR Database (http://qsar.food.dtu.dk/) collected estimates from over 200 QSAR models from free and commercial platforms, including descriptors like ecotoxicity, environmental fate, physicochemical properties, and ADMET. Trivedi et al. (2019, 2020) used the online tool Danish QSAR database to determine the ADMET properties for potential hits for Dengue fever and H1N1 flu, respectively. Multicase acute aquatic toxicity, carcinogenicity, arylhydroxylase activity, lethal body burden, bioconcentration, mutagenicity, biodegradation, environmental partitioning, and general properties are included in the ADMET properties. Finally, 12 compounds were identified as potential leads against dengue fever and 18 compounds against H1N1 flu. (2) The OCED Toolbox (https://www.oecd.org/chemicalsafety/risk-assessment/oecd-qsar-toolbox.htm), a package for toxicity prediction, was also developed based on QSAR. Han et al. (2019) used OECD QSAR Toolbox 4.1 to predict the genotoxicity for ceftazidime (CAZ) and its impurities to improve quality control of drugs. (3) ADMET Predictor^TM^ (https://www.simulations-plus.com/software/admetpredictor/) is another tool utilizing QSAR to predict ADMET parameters of compounds. Takac et al. (2019) used ADMET Predictor^TM^ to investigate the potential impact and safety profile with respect to the environment and health for 25 selected entactogen molecules. The chemical structure (including 1D and 2D) information was used as the input for ADMET Predictor^TM^. Lipophilicity parameters, volume of distribution, jejunal permeability, solubility, and logarithm of the brain/blood partition coefficient were predicted in this case. Alarn and Khan (2019) used the ADMET Predictor^TM^ to predict pharmacokinetics, pharmacodynamics, and toxicity parameters of flavone analogs to reveal their anticancer activity. Different physicochemical properties were calculated as descriptors to build the model, and then numerous properties were predicted, such as solubility, lipophilicity, permeability, absorption, bioavailability, BBB, transporters, plasma-protein binding, and volume of distribution.

Although the use of QSAR models has made considerable progress in ADMET prediction, these models cannot yet be used to replace *in vitro* or *in vivo* studies for all endpoints. The QSAR method is always limited by its model expansion capability, and large experimental data are always needed for model construction. The narrow data distribution may induce over fitting and lead to inaccurate prediction results. For example, Verheyen et al. (2017) estimated the QSAR models used for the prediction of eye and skin irritation/corrosion in Derek Nexus, Toxtree and Case Ultra. They found that the prediction results were unsatisfactory because of the narrow application range and low accuracy. Thus, validation and documentation for a constructed model is important prior to use.

#### PBPK Modeling

Most traditional models for predicting drug pharmacokinetics are empirical models. With a deeper understanding of the pharmacokinetic mechanism of drugs, PBPK models have been developed to predict PK properties (De Buck and Mackie, 2007). The PBPK model is an arithmetical model that combines drug data (e.g., drug concentration and clearance rate) and species physiology parameters to replicate the PK profile of a drug in plasma and tissues, aiming to describe *in vivo* drug pharmacokinetics that are related to tissue volume, administration routes, blood flow, biotransformation pathways, and interactions with tissues or organs in the body (Espie et al., 2009; Jones et al., 2015). The origin of the PBPK models can be traced back to Teorell's work in 1935. Teorell introduced a multicompartment model to simulate pharmacokinetics, which organically combined physiology and biology for the first time (Teorell, 1935; Zhao et al., 2011). Teorell's work has since attracted serious attention to the PBPK model.

In recent years, PBPK modeling has been substantially improved, making it more widely applicable for the research and development of drugs (Edginton et al., 2008; Rowland et al., 2011; Zhuang and Lu, 2016). Moreover, the increase in preclinical data, especially *in vitro* data, has promoted the development of PBPK models and simulations in drug discovery (Zhuang and Lu, 2016). PBPK modeling describes the physical and biological disposition of each compartment by dividing organisms into individual organs, and the most common processes are related to blood transportation and penetration, distribution between blood and organ tissue, and metabolic excretion, among others (Schmitt and Willmann, 2004). Since PBPK integrates large amounts of drug-specific data, parameters, and species physiology (systematic data), there are two kinds of parameters in PBPK models, which use the concentration-time curves of all organs and blood as output information (Nestorov, 2003). The first type consists of physiological parameters, such as tissue volume, blood flow, and cardiac output. Recently, due to the extensive application of *in vitro-in vivo* extrapolation (IVIVE) in PBPK, many researchers have predicted the disposal of drugs *in vivo* through *in vitro* metabolism and transport data, indicating that metabolic enzymes and transporter expression data have become important physiological parameters (Rostami-Hodjegan, 2012). The second type consists of drug-related parameters, such as the plasma ratio, organ/blood partition coefficient, and permeability (Nestorov, 2003). Recently, PBPK models have been widely constructed to predict drug-related parameters (Chow et al., 2016; Pathak et al., 2019; Song et al., 2020). For example, Chow et al. used a physiologically based model to predict drug solubility and effective permeability (Chow et al., 2016) to examine the potential impact of excipients on oral drug absorption.

## Databases

In the past 10 years, with rapid development, a number of related databases storing pharmacokinetic parameters have emerged. We collected some of the most commonly used databases and classified them as ADMET-related databases and auxiliary databases. A brief introduction to these databases, including website links, data scales, and descriptions, is provided in Tables 1, 2. For the ADMET-related databases, users can submit information on the compounds they want to query through the corresponding modules. Then, shape screening or pharmacophore screening will be performed to obtain additional targets or bioactivity information on similar ligands that match the query molecule. The ADMET-related properties can also be obtained from the query result. The auxiliary databases mainly focus on providing structural information about compounds. Although some ADMET-related information is provided in the search results, it is not complete, and not every compound is associated with such information.

**Table 1:** Some widely used ADMET-related databases.

**Database name**	**Availability**	**Description**	**URL**
ACD/Percepta	Commercial	Used to calculate PhysChem properties, ADME properties, and toxicity endpoints	https://www.acdlabs.com/products/percepta/index.php
ADME Database	Commercial	A commercial database for studying drug interactions and ADME, updated quarterly	https://www.fujitsu.com/jp/group/kyushu/en/solutions/industry/lifescience/admedatabase/
AurSCOPE ADME	Commercial	A complete annotated, structured knowledge base can be used to design predictive models and identify potential drug interactions	http://www.aureus-sciences.com/
Knowitall	Commercial	The world's largest mass spectrometry library, can be used to quickly and accurately identify the spectrum	http://www.bio-rad.com/
ToxBank	Commercial	A cross-cluster project can be used for comprehensive data analysis of toxicology and alternative detection of repeated dose toxicity tests	http://toxbank.net/
ACToR	Free	Exploring and visualizing complex computational toxicology information	https://actor.epa.gov/actor/home.xhtml
BRENDA	Free	Database of enzyme and enzyme-ligand information	http://www.brenda-enzymes.org/
CEBS	Free	A toxicological resource that can be used to model, predict analyze and assess effects of time and dose on responses to experimental conditions	https://manticore.niehs.nih.gov/cebssearch
ChemTunes	Free	A unique cheminformatics platform and expert QC'ed database that facilitate and support the safety and risk assessment process for chemical substances	https://www.mn-am.com/products/chemtunes
CTD	Free	A public resource storing scientific data about relationships between genes, chemicals, and human diseases	http://ctdbase.org/
DIDB	Free	Allows researchers to perform the assessment of human PK-based drug interactions and drug safety	https://www.druginteractioninfo.org/
DSSTox	Free	A public resource of chemical structure information with existing toxicity data for supporting better predictive toxicology	https://www.epa.gov/chemical-research/distributed-structure-searchable-toxicity-dsstox-database
eChemPortal	Free	Chemical substance search, chemical property data search and GHS search are available	http://www.oecd.org/chemicalsafety/risk-assessment/echemportalglobalportaltoinformationchemicalsubstances.htm
IDA2PM	Free	An integrated database for data access transformation and analysis visualization and prognostic modeling	http://idaapm.helsinki.fi/
Liceptor Database	Free	A ligand database containing 2D structures, related molecular descriptors and bioactivity data consisting of assays, functions and therapeutic hints	http://www.evolvus.com/
Metabolism and Transport Database	Free	A database related to small molecule transport metabolism that can be used for computational analysis and modeling	http://www-metrabase.ch.cam.ac.uk/
MetaCyc	Free	A database of metabolic pathways that can predict the metabolic pathways of sequenced genomes	https://metacyc.org/
NTP	Free	NTP studied at any chemical with the potential to impact health	https://sandbox.ntp.niehs.nih.gov/neurotox/
Pharmaco Kinetics Knowledge Base (PKKB)	Free	A free database that collects ADMET data and can be used for ADMET modeling	http://cadd.zju.edu.cn/pkkb/
Repdose	Free	A toxicity database storing *in vitro* and *in vivo* data and a prediction system for the safety and risk evaluation	https://repdose.item.fraunhofer.de/about_repdose.html
RTECS	Free	Contains additional information related to the chemical industry and occupational safety and health that can be used to assess workers' exposure to chemicals	https://www.atsdr.cdc.gov/substances/index.asp
SuperToxic	Free	Can perform similarity screening, risk assessment, and link to other databases	http://bioinformatics.charite.de/supertoxic/
T3DB	Free	Describe the relationship between toxins and targets and the mechanism of toxic action, which can be used for prediction of toxicity, prediction of toxic targets, etc.	http://www.t3db.ca/
TOXNET	Free	It is composed of a set of databases dealing with environmental health of toxicological hazardous chemicals and related fields	https://toxnet.nlm.nih.gov/
ToxRefDB	Free	Store data from *in vivo* animal toxicity tests and provide toxicity endpoints for predictive modeling	https://catalog.data.gov/dataset/toxcast-toxrefdb

**Table 2:** Some auxiliary databases for ADMET prediction.

**Database Name**	**Availability**	**Description**	**Scale**	**URL**
PubChem	Free	A public database storing biological properties of small molecules	Contains biological test results for more than 700,000 compounds	http://pubchem.ncbi.nlm.nih.gov/
DrugBank	Free	Storing information about drugs and related targets	Contains 13,490 drug entries	https://www.drugbank.ca/
STITCH	Free	A search tool and resource for interactions of chemicals and proteins	Interaction data for more than 68, 000 different chemicals	http://stitch.embl.de/
ChEMBLdb	Free	An open large bioactive database that can display metabolite pathways and link to metabolite metabolizing enzymes and information document source data	Containing more than 1.6 million individual compound structures denoted in the database, with 14 million activity values from more than 1.2 million assays	https://www.ebi.ac.uk/chembldb/
BindingDB	Free	A web-based resource containing experimental binding affinities, focusing mainly on the interactions between potential drug-targets and drug-like molecules	1,794,819 binding data, for 7,438 protein targets and 796,104 small molecules	http://www.bindingdb.org/bind/index.jsp
ChemProt	Free	A resource to perform *in silico* estimation of small molecules with the integration of molecular, cellular, and disease-related proteins complexes	over 1.7 million compounds with 7.8 million bioactivity measurements for 19,504 proteins	http://potentia.cbs.dtu.dk/ChemProt/
SIDER	Free	Contains information about marketed drugs and their adverse reactions, which can be used to quickly track trace the origin of an extracted side effect	5,868 side effect and 1,430 drugs and 139756 drug-SE pairs	http://sideeffects.embl.de/drugs/
MetaADEDB	Free	A comprehensive database of adverse drug reactions (adr), which is used to predict clinical adr and the source of adverse drug side effects of personalized drugs, and to predict drug interactions with targets and drug action patterns	3,060 chemicals (including more than 1,300 FDA approved and experimental drugs) and 13, 256 ADEs	http://lmmd.ecust.edu.cn/online_services/metaadedb/
TTD	Free	Provides data about known and studied therapeutic macromolecules, the targeted disease, pathway information, and the associated drugs directed at each of these targets	2,589 targets and 31,614 drugs	http://bidd.nus.edu.sg/group/cjttd/
KEGG	Free	A reference repository that is widely used to integrate and interpret large-scale datasets obtained by genome sequencing and other high-throughput experimental technologies	4 categories (systems, genomic, chemical and health information) from 18 databases	https://www.kegg.jp/

### ADMET-Related Databases

At present, many *in silico* models are used to predict ADMET, but massive amounts of data are needed to build them. The quality and quantity of the data are closely related to the accuracy of model prediction, so reliable experimental data are the key to successful prediction (Dearden, 2007; Alqahtani, 2017). Currently, there are some databases that can help ADMET prediction, such as the ADME Database (Shang et al., 2017), SuperToxic (Schmidt et al., 2009), PKKB (Cao et al., 2012), and DSSTox (Williams et al., 2017). By using these databases, users can obtain helpful data sets for use in external algorithms to generate prediction models. The databases can also be used directly to perform prediction through search functions, such as the similarity search or prediction. In addition, these models can be updated as new experimental data are added to the database.

The ADME Database (https://www.fujitsu.com/jp/group/kyushu/en/solutions/industry/lifescience/admedatabase/), developed by Zagreb University and Fujitsu in 2004, is a commercial database that specializes in pharmacokinetics information. It provides comprehensive data on drug-metabolizing enzymes and drug transporters that are specific to humans. The data have been widely used in drug research and development, such as ADME prediction and drug-drug interactions. Users can search for classification, metabolic reactions, and kinetics-related information about compounds by structure or substructure. However, the database currently limits large-scale downloads of user data, as well as public dissemination of some models.

SuperToxic collects toxins from different sources (animals, plants, synthetic, etc.), compiles ~60 000 compounds with their structures, and integrates some chemical properties and commercial availability information (Schmidt et al., 2009). These compounds are classified based on their toxicity, which derives from more than 2 million measurements. The values can be used to study the relationship between chemical structures and functions of toxins for evaluating the risk of their use. Users can easily query the structure and toxicity information of all compounds with corresponding properties through a structure search, name search, or property search. SuperToxic also allows users to browse the data by choosing an alphabetic character or numbers to present all entries starting with the selection (Schmidt et al., 2009). The available CASRN or NSC numbers in the database can also be recorded. The toxicity information retrieved from the database includes the dosage, type of test (toxicity measurement, such as LD50), and cell lines or organisms that determine the toxicity (Schmidt et al., 2009). The database also integrates software packages that are widely used in modern composite database construction, such as Marvin Sketch (molecule drawing), JMol (visual inspection), and MyChem/OpenBabel (property calculation). Furthermore, SuperToxic was connected to the Protein Data Bank, UniProt, and KEGG databases to identify potential targets in biochemical pathways to search for compounds (Schmidt et al., 2009).

The EPA Distributed Structure-Searchable Toxicity (DSSTox) database, which provides a series of documented, standardized and complete structure annotated toxicity information files, can be very useful for SAR model development (Richard and Williams, 2002; Williams et al., 2017). To allow wider use of the database, DSSTox was designed to use a structure data file (SDF), a public and industry-standard import/export file format storing chemical structures and property information that can be used as input for any chemical relational database (CRD) application or converted to data tables. It is one of the best-curated public datasets available at present, and the data stored in it are regarded as a standard reference for publicly available structural toxicity-based data.

Except for the databases introduced above, some newly constructed ADMET-related databases should also be of concern, for example, Comparative Toxicogenomics Database (CTD) (Davis et al., 2019), The Toxicity Reference Database (ToxRefDB) (Watford et al., 2019), and The Chemical Effects in Biological Systems database (CEBS) (Lea et al., 2017). CTD is a powerful and public database designed to enhance understanding of how environmental exposures influence human health. It provides data on chemical–gene/protein interactions, chemical–disease and gene–disease relationships that are combined with pathway and function data to help develop hypotheses about the inherent mechanisms of diseases affected by the environment (Davis et al., 2019). ToxRefDB collects data from *in vivo* animal toxicity tests and provides toxicity endpoints for predictive modeling. Approximately 28,000 datasets from nearly 400 endpoints have been generated and stored. The recent update of ToxRefDB has added connections to other resources and significantly enhanced the utility of predicting toxicology (Watford et al., 2019). CEBS offers a toxicology resource that compiles individual and concise animal data from 11,000 test articles and more than 8,000 studies encompassing all available National Toxicology Program (NTP) carcinogenicity, genetic toxicity, and short-term toxicity studies. The high-quality data in CEBS is very useful for constructing a more accurate model for toxicity prediction (Lea et al., 2017). We may infer from the recent constructed databases that the quantity and quality of the data determine the quality of the model and will be our focus in the future.

### Auxiliary Databases

In addition to ADMET-related databases, databases of biological activity, pathways, and side effects are important for ADMET prediction. Most of these databases are free and open to visitation, such as DrugBank (Wishart et al., 2018), PubChem (Kim et al., 2019), and ChEMBL (Gaulton et al., 2017). Although they are rarely used to predict ADMET-related properties directly, they can provide structural information to build models or be queried for information about compounds. Users can also download the predicted compound structure and use it as an input file for other software.

DrugBank, a comprehensive database, integrated thousands of well-studied drugs and drug targets with their physical, chemical, biological, and pharmaceutical data (Wishart et al., 2006, 2008). DrugBank 4.0 was further expanded to contain data on ADMET and other kinds of QSAR information (Law et al., 2013). DrugBank 5.0, the latest version, has further updated this information (Wishart et al., 2018). Users can use chemical shifts or mass-to-charge ratio (m/z) lists to search DrugBank's spectral library for approximate or exact matches. DrugBank also systematically classifies compounds into different types based on structural features and structural similarities and allows users to query it by using a simple text (Law et al., 2013).

Pubchem, a public database of small molecules with their biological properties, consists of three interconnected parts: (1) Compound, storing over 102 million unique chemical structures provided by various depositors; (2) Substance, containing more than 251 million records including complexes, extracts, mixtures, and non-characteristics; and (3) BioAssay, containing more than 1,067,000 bioassays, providing composite adjacent structures, substructures, similar structures, bioactivity data, and other search functions (Kim et al., 2019).

ChEMBL is an open data database containing two-dimensional structures, calculated properties (molecular weight, lipophilicity, etc.) and abstract biological activities (pharmacology and ADMET data) for numerous drug-like bioactive compounds (Gaulton et al., 2017). It is composed of three different datasets that were originally developed by Inpharmatica, including StARlite, CandiStore, and DrugStore (Overington, 2009). The data in ChEMBL were extracted from the scientific literature and designed to meet the needs of users to intelligently cluster relevant information and integrate data across therapeutic studies and areas.

## Software

**Figure 5:**
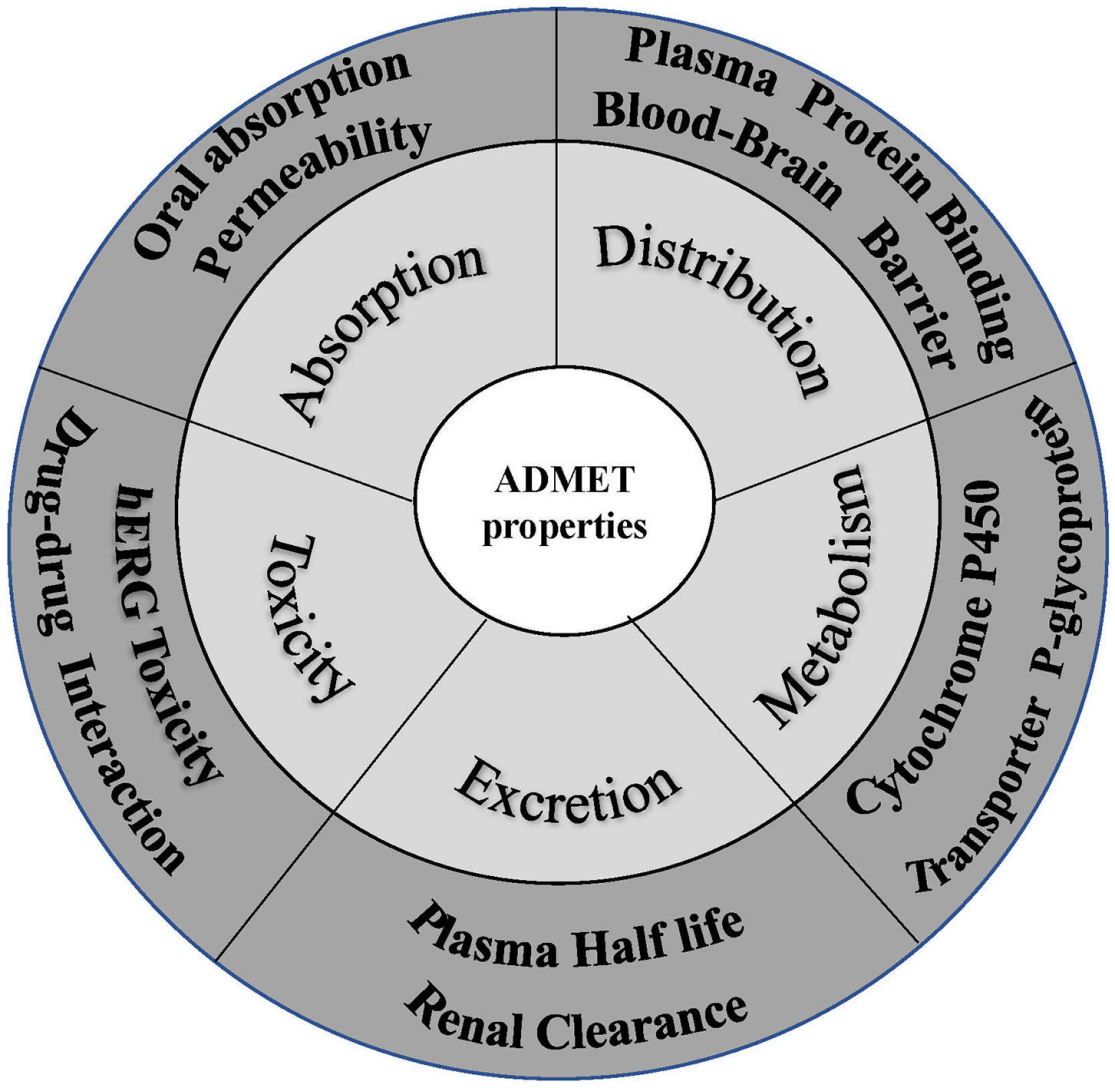
Crucial properties of ADMET.

Favorable ADMET characteristics are important as early requirements for drug candidates to reduce late failure and cost. However, many ADMET properties are highly dependent on each other, so they need to be optimized simultaneously in preclinical studies on drug discovery and development. Nevertheless, concurrent optimization of multiparameter ADMET is the most difficult and least attractive stage. As a result, early prediction of ADMET involved only some simple properties, such as logP, logD, and logS. With increasing experimental data, an increasing number of *in silico* models were developed to predict more complex ADMET parameters, such as the human intestinal absorption rate, oral bioavailability, blood-brain barrier permeation rate, Caco-2 permeability, human intestinal absorption, drug interactions, P-glycoprotein, plasma protein binding rate, CYP metabolic enzymes, and kidney clearance (Pires et al., 2015; Dong et al., 2018) (Figure 5). People have also attempted to integrate these models to predict ADMET parameters concurrently, and many studies have described these *in silico* models and their predicted properties (Dickins and van de Waterbeemd, 2004; Wang et al., 2015). In addition, software integrating these models to predict ADMET parameters concurrently has been developed. Tables 3, 4 list some of these software packages (free and commercial) with their functions. We also compared five commonly used software in Table 5 with each other to visualize their detailed functions.

**Table 3:** Some free ADMET software and related properties in their prediction.

**Software**	**URL**	**LogS**	**LogP**	**LogD**	**Sol**	**TPSA**	**HIA**	**Caco-2**	**PPB**	**BBB**	**V_d_**	**Met**	**CL**	**Tox**	**P-gp**	**per**	**pKa**	**MDCK**
ACD/I-lab	http://www.acdlabs.com/home/	√	√	√	√	√	√		√	√	√			√	√		√	
ADMETlab	http://admet.scbdd.com/	√	√	√	√	√	√	√	√	√	√	√	√	√	√			
admetSAR	http://lmmd.ecust.edu.cn/admetsar1/				√		√	√		√		√		√	√			
FAF-Drug4	http://fafdrugs4.mti.univ-paris-diderot.fr.		√			√												
Lazar	https://www.in-silico.de													√				
OCHEM	http://ochem.eu	√	√		√		√			√		√		√		√		
OECD Toolbox	http://toolbox.oasis-lmc.org/											√		√				
OSIRIS property explorer	http://www.organic-chemistry.org/prog/peo/	√	√		√	√								√				
pkCSM	https://smartcyp.sund.ku.dk				√		√	√		√	√	√	√	√	√	√		
SMARTCyp	https://nodepit.com/node/org.lhasalimited.knime.metabolism.encapsulated.smartcyp.SMARTCypNodeFactory											√						
SwissADME	http://www.swissadme.ch	√	√		√	√	√			√		√			√	√		
ToxCreate	https://github.com/opentox/toxcreate						√							√				
ToxTree	http://toxtree.sourceforge.net/#carousel0											√		√				
VCCLAB (ALOGPS 2.1)	http://www.vcclab.org	√	√	√	√												√	
vNN-ADMET	https://vnnadmet.bhsai.org/vnnadmet/login.xhtml									√		√		√	√			

*logS, aqueous solubility; LogP, octanol-water partition coefficient; LogD, octanol-water distribution coefficient; Sol, solubility; TPSA, topological polar surface area; HIA, human intestinal absorption; PPB, plasma protein binding; BBB, blood-brain barrier; V_d_, volume of distribution; Met, metabolism; CL, clearance; Tox, toxicity; P-gp, P glycoprotein; Per, Permeability; pKa, acidity coefficient; MDCK, madin Darby canine kidney cell line*.

**Table 4:** Some commercial ADMET software and related properties in their prediction.

**Software**	**URL**	**LogS**	**LogP**	**LogD**	**Sol**	**TPSA**	**HIA**	**Caco-2**	**PPB**	**BBB**	**V_**d**_**	**Met**	**CL**	**Tox**	**P-gp**	**Per**	**pKa**	**MDCK**
ACD/Percepta Platform	https://www.acdlabs.com/products/percepta/	√	√	√	√	√				√	√	√		√	√		√	
ADMEWORKS	https://www.fujitsu.com/jp/group/kyushu/en/solutions/industry/lifescience/admeworks/	√	√		√		√			√		√		√	√			
CompuDrug's Pallas System	http://www.compudrug.com/pallas_system		√	√		√						√		√			√	
Derek Nexus	https://www.lhasalimited.org/products/derek-nexus.htm													√				
MCASE, CASE, CASETOX	https://www.multicase.com/													√				
MetaSite	http://www.moldiscovery.com/soft_metasite.php											√						
PASS	http://genexplain.com/pass/											√		√				
Schrodinger QikProp	https://www.schrodinger.com/qikprop/	√	√	√	√			√		√				√				√
Simulations Plus ADMET Predictor	https://www.simulations-plus.com/software/admetpredictor/		√	√	√				√	√	√	√	√	√	√	√	√	√
StarDrop	https://www.optibrium.com/stardrop/stardrop-p450-models.php		√	√	√	√	√		√	√		√		√	√			
TIMES	http://oasis-lmc.org/products/software/times.aspx													√				
TOPKAT	http://www.moldiscovery.com/software/vsplus/													√				
VolSurf+	http://www.moldiscovery.com/software/vsplus/		√	√	√					√	√	√					√	

*logS, aqueous solubility; LogP, octanol-water partition coefficient; LogD, octanol-water distribution coefficient; Sol, solubility; TPSA, topological polar surface area; HIA, human intestinal absorption; PPB, plasma protein binding; BBB, blood-brain barrier; V_d_, volume of distribution; Met, metabolism; CL, clearance; Tox, toxicity; P-gp, P glycoprotein; Per, Permeability; pKa, acidity coefficient; MDCK, madin Darby canine kidney cell line*.

**Table 5:** Comparison of five commonly used ADMET software programs.

**Tools**	**Availability**	**Batch computation**	**Endpoints**	**Database**	**Druglikeness rules**	**Druglikeness model**	**Systematic evaluation**	**Medicinal chemistry friendliness properties**	**Physico- chemistry properties**	**Similarity**	**QSAR model**	**Algorithms**	**Training sets**	**Pattern recognition**
SwissADME	Free	√	Number, 19 Contents: B, A, D, M	×	√	×	√	√	√	√	×	×	×	×
ADMETlab	Free	√	Number: 31 Contents: B, A, D, M, E, T	√ (288,967 entries; 5 similarity searching strategies)	√ (5 rules)	√	√	×	√	√	√	√	√	×
admetSAR 2.0	Free	×	Number: 47 Contents: B, A, D, M, E, T	√ (210,000 entries)	×	×	√	×	√	√	√	√	√	×
Lazar	Free	×	Number: 11 Contents: T: Acute toxicity; BBB; Carcinogenicity, LOAEL, Maximum Recommended Daily Dose, Mutagenicity	×	×	×	×	×	×	√	√	√	√	√
ToxTree	Free	×	Number: 6 Contents: M, T	×	×	×	×	×	×	√	×	×	×	√

*B, basic physicochemical property; A, absorption; D, distribution; M, metabolism; E, excretion; T, toxicity*.

SwissADME is a hybrid web server that was developed by the Swiss Institute of Bioinformatics (Daina et al., 2017). It supports diverse input formats and can predict and analyze the ADME properties of numerous compounds in batches submitted from all over the world. This software outputs different types of physicochemical properties of drugs: water solubility, lipophilicity, physicochemical properties, druglikeness, pharmacokinetics, and medicinal chemistry, which can be directly exported and saved as a data file in CSV (comma-separated values) format and read by programs such as WordPad and Excel (Daina et al., 2017). In addition, it supplies a bioavailability radar map to quickly and intuitively evaluate the druglikeness of small molecules, facilitating its use for non-experts without professional knowledge (Daina et al., 2017). The server uses a variety of rules to evaluate the same property and provides the evaluation criteria and basis for most of the predicted data. However, in the prediction of whether the compound is a substrate or an inhibitor of the CYP enzyme, only a propensity is output, rather than a probability output similar to that of admetSAR. SwissADME also supplies a link for the one-click submission of the queried molecules to other servers in the Swiss series for further analysis (Daina et al., 2017).

Although *in vivo* toxicology is still the gold standard for identifying drug side effects, it is now believed that this method will not help reduce the large consumption rate in late clinical development (Merlot, 2010). Many computational tools have been developed to predict drug toxicity, helping to decrease the attrition rate of molecular compounds in drug discovery and reduce drug development time and cost. In recent years, the predictive power of these toxicology prediction systems has tremendously improved, covering more complex toxicological endpoints, such as hepatotoxicity, teratogenicity, nephrotoxicity, and carcinogenicity (Muster et al., 2008; Yang et al., 2018b). Currently, many commercial and free web-based toxicity predictors are available, such as Lazar (Maunz et al., 2013) and Toxtree (Patlewicz et al., 2008; Bhhatarai et al., 2016).

Lazar, developed by *in silico* toxicology GMBH, is a tool based on OpenTox (an integrated interface for an interoperable prognostic toxicology framework) to predict toxicological endpoints such as carcinogenicity, reproductive toxicity, and long-term toxicity (Hardy et al., 2010). It uses data mining algorithms to predict the toxicity of new compounds based on the experimental training data. Data sets with chemical structure and biological activity can be used as training data. Thus, Lazar can serve as a universal predictive algorithm for any biological endpoint if sufficient experimental data are available, so users no longer need to consider chemical, biological, or toxicological expertise but derive *in silico* models from statistical standards (Maunz et al., 2013). Users need only to input the structure of the compound, and Lazar will search the database for a series of similar compounds and corresponding experimental data, which will be used to construct a local QSAR model. The prediction results using the model will be displayed in a graphical interface, which provides structural features and compounds similar to the query compounds and toxicity properties for each fragment (Helma, 2006; Maunz et al., 2013).

Toxtree is a free software program that was commissioned by the European Chemicals Agency (ECB), the joint research center of the European Commission (Bhatia et al., 2015). It was originally designed to enable effective development of the Cramer decision tree. The latest version of Toxtree included additional projects, such as corrosion rules, BfR/SICRET skin irritation, and the Verhaar scheme, with a total of 14 functional modules (Bhatia et al., 2015). It includes physiochemical exclusion rules and structural alert inclusion rules, which are used to categorize compounds (Bhhatarai et al., 2016). Unlike Lazar, it has no training set. Its prediction is based on structural filters, so there is no applicability domain. It handles molecular structure information by using a decision tree model for risk assessment (Bhhatarai et al., 2016). Users can access it at http://toxtree.sourceforge.net/ to predict the toxicity of structures of interest.

ADMETlab is a platform for systematic ADMET estimation based on a comprehensive collection of ADMET databases (Dong et al., 2018). The platform includes four functional modules, which are used for drug similarity assessment (based on Lipinski's rule of five and the Druglikeness model), ADMET endpoint prediction, system evaluation, and database/similarity search (Dong et al., 2018). Among them, “ADMET prediction” is the main module used; the other three are auxiliary modules. Users can query one or more compounds with the platform by using the SMILES, uploading an SDF format file, or drawing the chemical structure using the embedded JME editor. After the compound is uploaded, the platform will use multiple pharmacokinetics models built by the different integrated data sets to make extensive predictions of the ADMET properties. Model prediction results are output in an interactive data table containing predicted values and structures. The software allows batch prediction, and users can apply the “drug similarity assessment” module to filter out compounds that are unlikely to be lead compounds or drugs, achieving the purpose of preliminary screening (Dong et al., 2018). Users must select one model to acquire results for one or multiple molecules, which is proper for screening compounds at specific endpoints, and the results will provide reasonable ADMET recommendations for each endpoint. Therefore, users can perform rapid screening of ADMET properties based on these independent specific prediction models and even further deliberately optimize the chemical structures of compounds, making them more likely to become drugs (Dong et al., 2018). Considering the very large amount of collected data and numerous constructed QSPR models, ADMETlab is currently one of the most comprehensive platforms used in ADMET prediction.

AdmetSAR is a free and comprehensive tool for ADMET property prediction (Cheng F. et al., 2012). The ADMET-related property data stored in AdmetSAR were collected from published literature. AdmetSAR also includes a searchable tool called ADMET-Simulator, which combines predictive and superior QSAR models in a toolbox based on chemical informatics and can predict ~50 ADMET endpoints. AdmetSAR enables users to easily search for ADMET properties by querying CASRN, the common name, or the structure (Yang et al., 2018a). The new version of admetSAR (version 2.0) mainly focuses on *in silico* prediction of chemical ADMET properties (Yang et al., 2018a). More than 40 predictive models trained by state-of-the-art machine learning methods were implemented in admetSAR. Four functions were developed: (1) customizable ADMET risk filters, (2) QSAR-based ADMET property prediction, (3) toxicity prediction for environmental chemicals, and (4) environmental hazard assessment. ADMETopt (Yang et al., 2018c) is a new module added in version 2.0 for lead compound optimization according to the predicted ADMET properties.

Except for ADMET software, an increasing number of PBPK software have been developed to perform systematics of the drug process in human body. The development of PBPK software has further promoted the use of PBPK modeling methods (Bouzom and Walther, 2008; Edginton et al., 2008; Perdaems et al., 2010). PBPK software was used to build and use the PBPK models, which can be useful for the estimation of pharmacokinetic parameters during the drug development process. At present, PBPK software can be roughly divided into two categories, the user customized software and user-friendly software. A brief introduction to the two types of software is provided in Table 6, including the company/institute and website links. In Table 7, we compare the functions of the commonly used software packages for both categories, such as WinNonlin (https://www.certara.com/) and GastroPlus (https://www.simulations-plus.com/).

**Table 6:** Two types of several PBPK software programs.

**Software**	**Company/Institute**	**URL**
**USER CUSTOMIZED SOFTWARE**		
Kinetica	Thermo Fisher Scientific Inc.	http://kineticadownload.com/Kinetica5.1-SP1/Default.asp
MATLAB-simulink	The MathWorks Inc.	https://www.mathworks.com/
NONMEN	ICON	https://www.iconplc.com/innovation/nonmem/
SAAM II	Washington University	https://tegvirginia.com/software/saam-ii/
WinNonlin	Certara USA Inc.	https://www.certara.com/software/phoenix-winnonlin/
**USER-FRIENDLY SOFTWARE**		
Cloe® PK	Cyprotex	https://www.cyprotex.com/insilico/
GastroPlus	Simulations Plus Inc.	https://www.simulations-plus.com/
Medici-PK	Computing in Technology	http://www.cit-wulkow.de/
PK—Sim	Bayer Technology Services	http://www.systems-biology.com/sb/
SimCYP	Certara USA Inc.	https://www.certara.com/software/simcyp-pbpk/

**Table 7:** Comparison of commonly used PBPK software programs for two categories.

**User customized software**	**Latest version**	**Modeling language**	**Auxiliary tool**	**Operation method**	**Model**
WinNonlin	WinNonlin v6.4	R	Phoenix® NLME™	Built-in options or a combination of graphics and text commands	Non-compartmental analysis (NCA)
NONMEM	NONMEM v7.3	FORTRAN	Wings for NONMEM, priana	Fully based on text	Two-compartment or three-compartment model
**User-friendly software**	**Company**	**Species**	**Routes of administration**	**Features**	**Application**
GastroPlus	Simulations Plus Inc.	Human, rat, dog, mouse, monkey, user defined	i.v., p.o., ocular, pulmonary, lingual, sublingual, buccal	ACAT model PEAR function	Ten modules for PBPK modeling, built-in multi-person physiological treatment model, IVIVC; single simulation, batch simulation, parameter sensitivity analysis (PSA), population simulation
SimCYP	Certara USA Inc.	Human, rat, mouse, dog	i.v., p.o., pulmonary, skin	ADAM model, Extensive data base with physiological information	PBPK modeling function, IVIVE; DDI simulation and population differential prediction; time course of simulated metabolites
PK-Sim	Bayer Technology Services	Human, rat, mouse, dog, monkey, minipig	i.v., p.o., subcutaneous, dermal, user defined	WB-PBPK model, various PBPK calculation methods	High-flexibility-all model parameters accessible for specific investigations; Simulating physiological variability in reactions

It was initially used in engineering and mathematics of the user customized software because the essence of PBPK modeling is mathematical modeling (Bouzom et al., 2012). Hence, the application of these software programs in PBPK modeling is a natural transformation. The user customized software requires users to write their own programs to build the PK model at the beginning stage of development. This procedure requires users to have proficient programming skills as well as expertise in the field. Recently, specific PK or PBPK modules and equation libraries, as well as visual graphical interfaces, have been added to some of these software programs (Bouzom et al., 2012). By using these software programs, users can quickly generate standard PBPK models by following existing templates that already contain standard codes and equations, greatly facilitating user operation.

The user-friendly type, customized for PBPK modeling, has a graphical interface. It requires no modeling language and programming, so it is relatively simple to operate. Originally, some such software programs were specifically modeled for predicting a specific property of the ADME process, such as absorption (GastroPlus, Tian et al., 2011) or metabolism (SimCYP, Jamei et al., 2009). These software programs gradually evolved into complex PBPK modeling tools for the entire body. Recently, the function of updated versions has become increasingly sophisticated. Now, they not only model their specific areas but also simulate the whole-body pharmacokinetic process, which is absorption, metabolism, and excretion, etc. (Li M. et al., 2017; Byun et al., 2020). These software programs can perform various tasks, such as simulation, parameter evaluation, and sensitivity analysis, simply by inputting specific drug parameters and choosing certain model options (Bouzom et al., 2012).

## Applications

The *in silico* applications of predicting ADMET profiles in 2016-2018 were collected by searching PubMed. We analyzed the search results and briefly introduce how software programs and methods predict the properties of ADMETs.

### Molecular Modeling

Most applications of molecular modeling focus on predicting the strength of the interaction between a molecule and a metabolic enzyme or transporter. For instance, Niu et al. (2016) performed docking studies of flavokawain A (FKA) and its target CYP450. FKA shows obvious inhibition of different CYP isoforms, and subsequent inhibition experiments showed that CYP3A2 was the primary isoform contributing to the metabolism of FKA. Gong et al. (2018) performed a molecular docking experiment to study the binding mode between sauchinone and CYPs. The results showed the interactions of sauchinone in the active site of CYP2B6, 2C19, 2E1, and 3A4. In addition to the above examples, the details of 22 representative studies are included in Table 8.

**Table 8:** Applications of molecular modeling in predicting metabolic properties from 2016 to 2018.

**Year**	**Compound**	**Metabolizing enzyme**	**Tool**	**References**
2016	4 Steroid derivatives	CYP1B1 (3PM0)	Gold-5.4	Poirier et al., 2016
2016	8 omeprazole-based analogs	CYP2C19 (4GQS)	GOLD 5.2	Li et al., 2016
2016	Flavokawain A	CYP2D6 (3QM4)	SYBYL-X2.0	Niu et al., 2016
2016	Ketoconazole, Resveratrol, MAR, DAR and TAR	CYP3A4 (2V0M)	Discovery Studio 4.0	Basheer et al., 2016
2016	Naringenin, 6',7'-dihydrox-ybergamottin	CYP1A1 (4I8V)	Autodock	Santes-Palacios et al., 2016
2016	Pyridine, Piperidine and Azol scaffolds	UBE2D4	Discovery Studio 3.5	Ramatenki et al., 2016
2016	Rab38 inhibitors	Rab38	Discovery Studio 4.0	Abdelmonsef et al., 2016
2017	1′-S-1′-acetoxychavicol acetate	CYP1A2 (2HIF), CYP2D6 (3QM4), CYP3A4 (4D6Z)	AutoDock Vina version 1.1.2	Haque et al., 2017
2017	12 estrone (E1), 17 β-estradiol (E2) derivatives	CYP1B1(3PM0)	GOLD 5.4	Dutour et al., 2017
2017	Progesterone (PGS)	CYP3A4 (1W0F)	Schrödinger Suite, 2012	Du H. et al., 2017
2017	Quinoxaline, Diazepine, Piperazine	UBE2NL enzyme	LigPrep version 5.6, Schrödinger	Ramatenki et al., 2017
2017	Resveratrol, Nitrostilbene, Dimethoxy-nitrostilbene, Ketoconazole	CYP3A4(2V0M)	Discovery Studio 4.0 (CDOCKER), Schrodinger Suite 2016 platform (Glide docking)	Basheer et al., 2017
2017	Sulfonyl hydrazones	MAO-A and B	AutoDock Vina	Abid et al., 2017
2017	Wilfortrine, Wilforine, Wilfordine, Euonymine, Wilforgine	CYP3A4 (1W0F)	Discovery Studio (CDOCKER)	Wang L. et al., 2017
2017	XIAP	Caspase-3	AutoDock Vina	Prokop et al., 2017
2017	α-Naphthoflavone (ANF) 7-ethoxyresorufin (7ER)	CYP1A2 (2HI4)	GOLD 5.2.2	Watanabe et al., 2017
2018	15 Vinca derivatives	CYP3A4 (3NXU), CYP3A5	GOLD 5.2	Saba and Seal, 2018
2018	3 Compounds	CYP3A4 (4NY4)	Schrödinger Release 201702	Vaz et al., 2018
2018	Bavachin^a^, Neobavaisoflavone^b^, Corylifol A^c^	CYP1A2 (2HI4)^a, b, c^, CYP2C9 (1R9O)^a, b, c^, CYP2C19 (4GQS)^a, b, c^, CYP2D6 (3TGB)^a, b, c^, CYP3A4 (1WOF)^a, b, c^, CYP2E1 (3T3Z)^a, b, c^	Discovery Studio 4.1 (CDOCKER)	Wang L. et al., 2018
2018	FAK and Triazinic inhibitors	FAK	Schrödinger 9.0	Cheng P. et al., 2018
2018	Metconazole (MEZ) isomers	CYP3A4 (2V0M)	AutoDock Vina	Zhuang S. et al., 2018
2018	Paracetamol^a^, Pilocarpine^b^	CYP2E1 (3T3Z)^a,b^, CYP3A4^a^	SYBYL-X 1.3	Wang Y. et al., 2018
2018	Sauchinone	CYP3A4 (3UA1), CYP2B6 (3IBD), CYP2C19 (4GQS), CYP2E1 (3GPH)	AutoDock 4.2.6	Gong et al., 2018
2018	Sulfaphenazole, Chondroitin disaccharide Δdi-4S (C4S), Glucosamine 3-sulfate, Glucosamine 6-sulfate, Diacerein, Rhein	CYP2C9 (1R9O)	Discovery Studio 4.0 (CDOCKER)	Tan et al., 2018

*Superscript values denote that the metabolizing enzymes in the second column correspond to the query molecules in the first column, respectively*.

### QSAR

Prediction of the pharmacokinetic properties of compounds using QSAR relies mainly on traditional models or software developed based on constructed data sets. Table 9 lists seven typical applications of the QSAR method. For example, Khan et al. (2016) utilized the QSAR model in ACD/I-lab to determine multiple ADMET properties (such as logS, logP, logD, BBB) for 6 compounds targeting heat-shock protein 90 (Hsp90). Then, six compounds were designed according to BBB and antiangiogenic properties. One molecule (compound 6) was observed to inhibit Hsp90 with a predicted efficiency of BBB permeation of 0.55 kcal/mol in comparison to the experimental value of 0.625 kcal/mol. Ajay Kumar et al. (2018) performed 3D-QSAR studies to filter compounds based on ADME properties by using Schrodinger. Fifty hit compounds targeted to transforming growth factor-β (TGF-β) type I were screened based on predicted ADME properties (such as BBB, logS, and Lipinski's rule of five). Seven molecules were finally selected as the lead compound for subsequent research.

**Table 9:** Applications of QSAR in predicting ADMET properties from 2016 to 2018.

**Year**	**Tool/Method**	**Compound**	**Properties**	**References**
2016	ACD/I-lab	Angiogenic inhibitor for brain tumor	logS, logP, logD, logBB, hERG inhibition, HBA, HBD, MW	Khan et al., 2016
2016	QikProp 4.6	N-pyridyl, Pyrimidine benzamides	logS, logBB, MDCK, logKP, metab, CNS, loghERG, HOA	Malik et al., 2016
2017	ACD ChemSketch	Flavonoids	MW, IC50, Index of refraction, Surface tension, Density, Polarity, logP	Das et al., 2017
2017	CoMFA model	CITCO, α-naphtholphthalein, diethylstilbestrol, TPP, phenytoin, (R)-ethotoin, (S)-ethotoin	Drug-drug interactions, logP	Kato et al., 2017
2017	Discovery Studio v3.5	Novel dibenzofuran derivatives	PSA, Solubility, HIA, Cytochrome P450 2D6, BBB, PPB, Hepatotoxicity	Ma et al., 2017
2017	Self-organizing molecular field analysis (SOMFA)	Curcumin analogs	MW, IC50, logP	Verma and Thareja, 2017
2018	CoMFA and CoMSIA	Scopoletin Phenolic Ether Derivatives	LC50, TPSA	Luo et al., 2018
2018	MLR, SYBYLX v1.3, CoMFA and CoMSIA	Amyloid β aggregation inhibitors	IC50	Aswathy et al., 2018
2018	Quantum Mechanics/ Molecular Mechanics (QM/MM)	DCHA inhibitors	IC50	Kollar and Frecer, 2018
2018	QikProp 4.6	TGF-type I inhibitors	IC50, BBB, logS	Ajay Kumar et al., 2018

*LogP, octanol-water partition coefficient; LogD, octanol-water distribution coefficient; logS, aqueous solubility; logBB, blood-brain barrier; hERG, human ether-a-go-go related gene block; HOA, human oral absorption; PPB, plasma protein binding; MW, Molecular weight; LC50, median lethal concentration; TPSA, topological polar surface area; PSA, polar surface area; IC50, half maximal inhibitory concentration; HIA, human intestinal absorption; MDCK, madin Darby canine kidney cell line*.

### PBPK

PBPK modeling has been consistently performed to predict pharmacokinetics with the help of some widely used software programs (Table 7). Seventy-four applications using three software packages are listed in Table 10. WinNonlin is one of the most widely used software programs in the prediction of pharmacokinetics. For example, Gestrich et al. (2018) used WinNonlin (v6.4) to analyze compartmental and non-compartmental gentamicin plasma concentrations vs. time. The peak drug concentrations and AUCs in young adults and older alpacas were compared, and both were significantly lower in young adults than in geriatric alpacas. The increased drug exposure and decreased clearance in geriatric alpacas created a greater risk of ADRs and/or therapeutic failure. Another software, NONMEM, is also widely used to predict the impact of drugs on the target population. It is the “gold standard” software package for analysis of population PK/PD data. For example, Polepally et al. (2018) used non-linear mixed-effects modeling in NONMEM (version 7.3) to analyze concentration-time data to estimate the effect of age on the pharmacokinetic parameters of lamotrigine (LTG). By comparing the pharmacokinetic characteristics of young adult and elderly epilepsy patients, it was concluded that the bioavailability of LTG was not affected by age (Polepally et al., 2018). However, LTG CL in the elderly was 27.2% lower than in young epilepsy patients. These findings are very useful for clinicians to offer optimal epilepsy care and support to elderly patients starting low-dose treatment (Polepally et al., 2018).

**Table 10:** Applications of PBPK in predicting ADMET properties from 2016 to 2018.

**Year**	**Tool**	**Compound /Preparations**	**Properties**	**References**
2016	GastroPlus	Alectinib	Oral bioavailability, Aqueous solubility, C_max_, AUC	Parrott et al., 2016
2016	GastroPlus	Bisoprolol, Nifedipine, Cimetidine, Furosemide	Oral absorption	Hansmann et al., 2016
2016	GastroPlus	Carvedilol loaded nanocapsules (CLN)	AUC, C_max_	George et al., 2016
2016	GastroPlus	Ketoconazole, Erythromycin	AUC, C_max_	Boetsch et al., 2016
2016	GastroPlus	Levofloxacin	logP, Plasma protein binding, AUC, C_max_	Zhu et al., 2016
2016	GastroPlus	Met XR 1000 mg tablets	Dose, logD, AUC, C_max_	Chen W. et al., 2016
2016	PKSim	Azathioprine	Hepatotoxicity	Thiel et al., 2016
2016	PKSim	Morphine and Furosemide	Vss value	Schlender et al., 2016
2016	WinNonlin	6-chloro-9-nitro-5-oxo-5H-benzo-(a)-phenoxazine (CNOB)	AUC, V_d_, CL, half-life	Wang J. H. et al., 2016
2016	WinNonlin	Arbekacin	V_d_	Hagihara et al., 2016
2016	WinNonlin	Busulfan (BU)	AUC, apparent clearance	de Castro et al., 2016
2016	WinNonlin	Cetuximab (CTX), Capecitabine (CCB)	C_max_, AUC, V_d_	Rachar et al., 2016
2016	WinNonlin	Glibenclamide	CL, V_d_, half-life	Rambiritch et al., 2016
2016	WinNonlin	Imatinib mesylate	Bioavailability, T_max_, C_max_	Arora et al., 2016
2016	WinNonlin	Midazolam	C_max_, V_d_	Vuu et al., 2016
2016	WinNonlin	Midazolam, Irinotecan	Clearance rate	Lee et al., 2016
2016	WinNonlin	Perfluorooctanoic acid (PFOA), Perfluorooctanesulfonic acid (PFOS), Perfluorohexane sulfonic acid (PFHxS)	AUC, renal clearance, C_max_, V_d_	Kim et al., 2016
2016	WinNonlin	Propofol	Plasma concentrations, V_d_	Chen J. Y. et al., 2016
2016	WinNonlin	Sodium succinate, Polysorbate, Arginine, Phosphate-buffered saline (PBS)	CL, Half-life, C_max_	Gupta et al., 2016
2016	WinNonlin	Tacrine hydrochloride	Skin penetration	Patel et al., 2016
2016	WinNonlin	Tilmicosin	AUC, C_max_	Zhang et al., 2016
2016	WinNonlin	Treosulfan (TREO)	C_max_, AUC	Romanski et al., 2016
2017	WinNonlin	Tacrolimus	Plasma clearance	David-Neto et al., 2017
2017	GastroPlus	Basmisanil	logD, Solubility	Yang et al., 2017
2017	GastroPlus	Buagafuran	Plasma Protein Binding, logP	Yang et al., 2017
2017	GastroPlus	Compound A (CPD A)	Bioavailability, C_max_, AUC	Stillhart et al., 2017
2017	GastroPlus	Mangiferin	Aqueous solubility, logD, logP, Permeability	Khurana et al., 2017
2017	PKSim	Cefazolin, Cefuroxime, Cefradine	CL	Dallmann et al., 2017
2017	PKSim	Endogenous IgG	CL, V_d_	Niederalt et al., 2017
2017	PKSim	Fentanyl, Alfentanil, Thiopental, Omadacycline, Amiodarone, Propylthiouracil	Plasma concentration	Pilari et al., 2017
2017	PKSim	Vorinostat	Dose	Moj et al., 2017
2017	WinNonlin	Acetylkitasamycin	C_max_, T_max_, AUC	Nan et al., 2017
2017	WinNonlin	Benznidazole	C_max_, AUC	Molina et al., 2017
2017	WinNonlin	Ceftiofur	CL, V_d_	Wang J. et al., 2017
2017	WinNonlin	Cloxacillin	Dose	Burmanczuk et al., 2017
2017	WinNonlin	Danofloxacin	AUC	Zhang N. et al., 2017
2017	WinNonlin	Diaveridine	Oral bioavailability, C_max_, AUC	Li Y. F. et al., 2017
2017	WinNonlin	Enrofloxacin	Plasma concentration	Shan et al., 2017
2017	WinNonlin	Iohexol	CL	Zhang C. et al., 2017
2017	WinNonlin	Lurasidone	C_max_, AUC	Hu et al., 2017
2017	WinNonlin	Meropenem	Plasma concentration	Kong et al., 2017
2017	WinNonlin	Metolazone	AUC, C_max_, T_max_	Li X. et al., 2017
2017	WinNonlin	Psilocybin	Nephrotoxicity	Brown et al., 2017
2017	WinNonlin	Pyrazinamide	C_max_	Voelkner et al., 2017
2017	WinNonlin	Sarafloxacin	Dose	Yu et al., 2017
2017	WinNonlin	Sildenafil	CL, T_max_, V_d_	Olguin et al., 2017
2017	WinNonlin	Tenofovir	CL, Plasma concentration	Du X. et al., 2017
2017	WinNonlin	Tilmicosin	AUC, T_max_, half-life	Zhang L. et al., 2017
2017	WinNonlin	Treosulfan	Liver, Brain toxicity	Romanski et al., 2017
2017	WinNonlin	Tulathromycin	Dose	Zhou et al., 2017
2018	GastroPlus	Cefadroxil	Permeability, logP, Aqueous solubility, Distribution volume	Hu and Smith, 2018
2018	GastroPlus	Compound-A	Bioavailability, logP, Permeability, Aqueous solubility	Kou et al., 2018
2018	GastroPlus	Dasatinib	AUC, C_max_, T_max_	Vaidhyanathan et al., 2018
2018	GastroPlus	DPP-4 inhibitors	Bioavailability	Daga et al., 2018
2018	GastroPlus	Lanabecestat (AZD3293)	Bioavailability	Ye et al., 2018
2018	GastroPlus	Tramadol	Renal clearance	T'Jollyn et al., 2018
2018	PKSim	Carvedilol	Plasma concentration	Ibarra et al., 2018
2018	PKSim	Escitalopram	Plasma exposure	Delaney et al., 2018
2018	PKSim	Indomethacin, Felodipine	Plasma exposure	Keemink et al., 2018
2018	PKSim	Pregabalin	Plasma concentrations	Idkaidek et al., 2018b
2018	WinNonlin	Cefquinome	C_max_, AUC	Shan et al., 2018
2018	WinNonlin	Dexmedetomidine	CL, V_d_	Song et al., 2018
2018	WinNonlin	Gentamicin	AUC	Gestrich et al., 2018
2018	WinNonlin	Gliclazide	V_d_, CL	Shaik et al., 2018
2018	WinNonlin	Letrozole	Dose	Arora et al., 2018
2018	WinNonlin	Moxidectin	CL, T_max_, C_max_	Xiao et al., 2018
2018	WinNonlin	Penicillin G	V_d_, CL, half-life	Padari et al., 2018
2018	WinNonlin	Seroquel XR, Quesero XR	AUC, C_max_, CL	Huang et al., 2018
2018	WinNonlin	Sitagliptin	C_max_, T_max_, AUC, T_1/2_	Sangle et al., 2018
2018	WinNonlin	Vitacoxib	Plasma concentrations, C_max_, AUC	Wang et al., 2018a
2018	WinNonlin	Amoxicillin/clavulanic acid tablets	C_max_, T_max_, AUC, T_1/2_	De Velde et al., 2018
2018	WinNonlin	Phenylbutyric acid (PBA), phenylacetic acid (PAA), and phenylacetylglutamine (PAGN), UPAGN	Plasma concentrations, AUC, T_max_	Berry et al., 2018
2018	WinNonlin	Siponimod	T_1/2_	Jin et al., 2018
2018	WinNonlin	Lacosamide	T_1/2_, V_d_, C_max_, CL, AUC	Franquiz et al., 2018
2018	WinNonlin	Tildipirosin (TD)	C_max_, AUC, T_max_	Wang et al., 2018b
2018	WinNonlin	Streptomycin	T_1/2_, AUC, T_max_, C_max_	Chen and Gao, 2018
2018	WinNonlin	Pantoprazole	T_1/2_, AUC, V_d_/F, CL/F	Shakhnovich et al., 2018
2018	WinNonlin	FVIII concentrates	T_1/2_	Cheng X. et al., 2018
2018	WinNonlin	Adherence to tenofovir disoproxil fumarate/emtricitabine (TDF/FTC)	C_max_, AUC	Ibrahim et al., 2018
2018	WinNonlin	Valsartan, Hydrochlorothiazide (HCT)	C_max_, T_max_, AUC, T_1/2_, K_el_	Idkaidek et al., 2018a
2018	WinNonlin	Individual total serum cortisol, unbound serum cortisol and salivary cortisone	C_max_, AUC, T_max_, Bioavailability	Johnson et al., 2018

*LogP, octanol-water partition coefficient; LogD, octanol-water distribution coefficient; AUC, area under the curve; C_max_, peak concentration; Vss, steady-state; V_d_, volume of distribution; T_1/2_, half-life; logP, octanol-water partition coefficient; logD, octanol-water distribution coefficient; CL, clearance; CL/F, body clearance corrected for bioavailability; V_d_/F, volume of distribution corrected for bioavailability. K_el_, elimination rate constant; F, bioavailability; T_max_, time to maximum concentration*.

GastroPlus and SimCYP are the mainstream PBPK emulation software programs. In recent years, applications have shown a growing trend in the use of these software programs along with the improvement of software functions. For example, Ye et al. (2018) constructed an absorption model using GastroPlus to predict the potential effects of different gastric pH levels on the pharmacokinetics of lanabecestat and found that changes in gastric pH had a minimal influence on clinical exposure to lanabecestat. They also compared the bioavailability of two tablet formulations and an oral solution. The results showed that the 90% confidence intervals for geometric mean ratios were within standard bioequivalence boundaries for all other pharmacokinetic parameters, indicating that both tablet formulations were located in the accepted bioequivalence criteria compared with the oral solution (Ye et al., 2018). Boland et al. (2018) used SIMCYP to generate a dose-concentration model by using data from different genders, ages, and oral morphine formulations. The model was then validated against clinical pharmacokinetics data and used to calculate the association of the morphine dose with the plasma concentration. Finally, the analysis showed that older age, female sex, modified-release formulation, and inferior renal function were related to higher plasma concentrations (Boland et al., 2018). This result can help clinicians provide personalized prescription decisions.

### ADMET

We listed ADMET prediction applications by using AdmetSAR, SwissADME, Lazar, or Toxtree in Table 11. For example, Petrescu et al. (2019) used the AdmetSAR computational program to study the cytotoxicity of 15 phenolic compounds. The results showed that these compounds were much less toxic to aquatic life than synthetic pesticides. Roman et al. (2018) used SwissADME, FAFDrugs4, and admetSAR to predict the ADMET profiles and pharmacokinetics of 31 anabolic and androgen steroids in humans. The results revealed that the investigated steroids showed high gastrointestinal absorption and good oral bioavailability, which may be useful in the inhibition of human cytochromes associated with the metabolism of xenobiotics. In addition, the side effects of the studied steroids in humans were also predicted. Silva et al. (2019) predicted the theoretical toxicity of fluconazole (FNZ) by using Lazar to study the toxicity profile of FNZ toward human peripheral blood mononuclear cells (PBMCs) cultured *in vitro*. The results showed that FNZ had potential mutagenic, tumorigenic, stimulating, and carcinogenic effects (Silva et al., 2019). Zhuang J. et al. (2018) used Toxtree (version 2.6.13) to evaluate the toxicity of extractables from multilayer coextrusion bags, and their prediction results revealed the types of extractables as well as the bioaccumulation factor and mutagenicity.

**Table 11:** Applications of predicting ADMET properties using software from 2016 to 2018.

**Year**	**Tool**	**Compound/Preparations**	**Properties**	**References**
2016	admetSAR	GSK-3 targeting ligands	Mutagenicity, BBB, HIA, Caco-2, MDCK, PPB, AMES test, carcinogenicity, rat acute toxicity, P-gp substrate/inhibitor probability	Nisha et al., 2016
2016	Lazar	A dataset of air toxins (332 chemicals), A subset of the gold carcinogenic potency database (480 chemicals)	Carcinogenicity, mutagenicity	Pradeep et al., 2016
2016	Lazar	The Schiff bases of Benzothiazol-2-ylamine, Thiazolo [5, 4-b] pyridin-2-ylamine	Max, daily dose, acute toxicity, LC50	Shukla et al., 2016
2016	ToxTree	Cinnamaldehyde, Eugenol	Biodegradability, genotoxicity, carcinogenicity, bioaccumulation, developmental toxicity, mutagenicity, LD50, LC50	Absalan et al., 2016
2016	ToxTree	PC-replacement products—the 48 substances chosen based on the publications of (Simoneau et al., 2012) and (Onghena et al., 2014, 2015)	Genotoxic carcinogenicity	Mertens et al., 2016
2016	ToxTree	Vaccine constituents	Carcinogenicity, mutagenicity, genotoxicity, LD50	White et al., 2016
2017	admetSAR	3-bromopyruvate, Dibromopyruvate (DBPA), Propionic acid (PA)	BBB, Human intestinal absorption, Caco-2 permeability, P-glp substrate, AMES toxicity, Acute oral toxicity, Acute toxicity, CYP450 substrate and inhibitor, hERG	Yadav et al., 2017
2017	admetSAR	Histone deacetylase (HDACs) inhibitors	LogS, Caco-2 permeability	Uba and Yelekci, 2017
2017	admetSAR	Vernonia anthelmintica (L.)	Bioavailability, HIA, Caco-2, metabolism CYP	Wang J. Y. et al., 2017
2017	Lazar	2-amino6-methylpyridine, 6-heptenoic acid, 2-methylphenol	Carcinogenicity, mutagenicity	Frenzel et al., 2017
2017	Lazar, ToxTree	8 volatile organic compounds (VOC)	Carcinogenicity, mutagenicity	Guerra et al., 2017
2017	SwissADME	3,7-dimethyl-2,6-octadienal, 2-pentene-2-methyl	Physicochemical properties, lipophilicity, hydrophilicity	Simhadri Vsdna et al., 2017
2017	SwissADME	338 different chemical pesticides	Lipophilicity, TPSA, molar refractivity, BBB permeant, GI absorption	Chedik et al., 2017
2017	SwissADME	Ginger	GI absorption, BBB, skin permeability, P-gp substrate	Sanni and Fatoki, 2017
2017	SwissADME	Polyphenols	Lipophilicity, water solubility	Yugandhar et al., 2017
2017	SwissADME	Tributyltin (IV) complex carboxylic acid derivative	GI absorption, BBB, LogP, water solubility, GI, Caco-2 Cells, Ames Test	Waseem et al., 2017
2017	SwissADME	Xeronine	Lipophilicity, GI absorption, solubility, bioavailability	Sanni et al., 2017
2017	ToxTree	400 compounds	skin/eye irritation, corrosion	Verheyen et al., 2017
2017	ToxTree	80 commercially available chemicals (38 liquids and 42 solids)	Eye irritation, corrosion	Geerts et al., 2017
2018	Lazar	(-)-Asimilobine, Aloin, Annoretine, Chrysothrone, Coptisine, Elymoclavine, Thalicminine	Genotoxicity, carcinogenicity, mutagenicity	Gluck et al., 2018
2018	Lazar	Newly proposed heterocyclic derivatives	Carcinogenicity, mutagenicity	Azad et al., 2018
2018	Lazar	The synthesized 1,3,5-trisubstituted−2-pyrazoline derivatives (5a-5t)	Maximum recommended daily dose, reproductive toxicity, carcinogenicity, Mutagenicity, Acute toxicity, LC50	Tripathi et al., 2018
2018	SwissADME	21 Organosilicone compounds	TPSA, logP, GI absorption, BBB	Shaaban et al., 2018
2018	SwissADME	Mycotoxins (DON,3-AcDON,15-AcDON)	HIA, BBB penetration, Mutagenicity, Carcinogenicity, Acute toxicity	Taroncher et al., 2018
2018	SwissADME	NAZ2329	Lipophilicity, Water Solubility, GI absorption, BBB permeant	Agoni et al., 2018
2018	SwissADME, admetSAR, ToxTree	31 anabolic hormones and androgen hormones	Gastrointestinal absorption, Blood brain barrier, P-gp substrate, Skin permeability, Carcinogenicity, hERG, Ames toxicity	Roman et al., 2018
2018	ToxTree	48 selected sensitizing and non-sensitizing AS	Skin sensitization	Braeuning et al., 2018
2018	ToxTree	Bis [2,4-bis(2-methyl-2-propanyl) phenyl] hydrogen phosphate	LD50, Bioaccumulation factor, Mutagenicity	Zhuang J. et al., 2018
2018	ToxTree	Printed paper and board FCM substances	Ames/bacterial mutagenicity	Van Bossuyt et al., 2018
2018	SwissADME, ToxTree	Dominant phytochemicals from Rheum palmatum, Rubus coreanus and Sanguisorba officinalis	logP, logD, TPSA, log S, GI absorption, CYP450 isoforms inhibitor probability, Genotoxicity	Nosrati et al., 2018
2018	SwissADME	8 mPGES-1 binders	PAINS	Lauro et al., 2018
2018	SwissADME	A new series of synthesized quinazoline derivatives	Molecular weight, logP, HBA, HBD, TPSA, Lipinski's RO5, Leadlikeness	Nasab et al., 2018
2018	SwissADME	107 Compounds containing biaryl scaffold	Molecular weight, logP, HBA, HBD, Lipinski's RO5, TPSA, BBB, GI absorption	Khalid et al., 2018
2018	SwissADME	Genistein, Daidzein and Glycitein	Lipinski's RO5, TPSA, Num. rotatable bonds	Shaji, 2018
2018	SwissADME	Four series of diphyllin-related compounds	LogP, PAINS	Lindstrom et al., 2018

*BBB, blood brain barrier; P-gp, P glycoprotein; HIA, human intestinal absorption; MDCK, madin Darby canine kidney cell line; PPB, plasma protein binding; GI absorption, gastrointestinal absorption; TPSA, topological polar surface area; LogP, octanol-water partition coefficient; LogD, octanol-water distribution coefficient; logS, aqeous solubility; F, Bioavailability; hERG, human ether-a-go-go related gene block; PAINS, pan-assay interference structures; LC50, median lethal concentration; LD50, median lethal dose; RO5, rule of five*.

## Discussion

### Deficiencies in Current *in silico* Methods

Each *in silico* method has its own characteristics and application scope. Hence, we need to select the most proper method for more accurate prediction. However, some methods have obvious deficiencies that may affect the prediction results. For example, molecular modeling plays a major role only in predicting metabolism and can assess only the possible interactions between compounds and metabolic enzymes; it cannot explicitly evaluate the ADMET risks of candidate compounds. The scoring function in molecular docking also affects the accuracy of ADMET prediction (de Graaf et al., 2006; Zhou et al., 2006). For instance, Kemp et al. (2004) and de Graaf et al. (2006) used different scoring functions to evaluate the binding affinities between cytochrome P450 and its inhibitors or substrates, respectively. Kemp et al. (2004) docked 33 compounds to P450, and the results revealed a correlation coefficient of *R*^2^ = 0.61 between the docking scores and active compounds. The docking scores were only able to identify several compounds as CYP2D6 inhibitors. Although de Graaf et al. (2006) integrated six scoring functions to identify the substrates of P450, the highest predicted accuracy (GOLD-Chemscore) identified 60% known substrates in the top 5% results during the virtual screening. Only high-affinity CYP2D6 ligands could be predicted. Therefore, docking methods with scoring functions are mostly applied for coarse screening of a series of compounds.

Compared with molecular modeling, data modeling can predict more properties, but its prediction accuracy depends on the quality and quantity of data. The QSAR method, the main strategy in data modeling, has limited value without an estimated model applicability domain for predicting biological or physicochemical properties (Sushko et al., 2010). The predictive ability of models will be limited if the predicted chemical is outside the chemical space where the models were developed (Sheridan et al., 2004). Furthermore, the descriptors used in the model construction for structural transformation are too simplistic and inadequate to predict the behavior of a drug in a whole organism. Therefore, there is a need to develop molecular descriptors containing more information. Many developed QSAR models have been validated only by internal validation without sufficient external validation, which is considered a necessary factor to build a reliable QSAR model (Roy et al., 2012). QSAR prediction is based on the principle that similar molecules have similar properties (Patterson et al., 1996), but in some special cases, such as CYP metabolism, similar molecules may have different activities, which are known as activity cliffs (Guha, 2011). Therefore, the ADMET properties of the compounds in the human body are not independent but are also affected by other factors. The PBPK method can predict multiple properties, but only provides common information about the biological behavior of organs or tissues. It is also limited by the mathematical form of the PBPK equations, which ignores the structural and physical properties of drug compounds (Huynh et al., 2009). Moreover, a large amount of experimental data is required when constructing models. Due to the lack of proper and easily accessible databases related to physiological properties, the data used to build models can be obtained only from the literature (Rowland et al., 2004). However, the obtained data are relatively limited, which reduces the predictability of the models. Finally, although some PBPK software programs have been developed, most of them are commercial, and users must participate in the training of software companies to make them more useful (Lave et al., 2007).

The existing ADMET software can perform faster and more convenient predictions of multiple properties to obtain more comprehensive prediction results. However, we can see in practical applications that the software is applicable only for qualitative analysis of compounds and cannot accurately predict the quantitative values of some properties. Moreover, issues are observed in the data quality and quantity of these software. While more experimental data are needed to further optimize the software, integrating unconfirmed data into the software to predict new compounds will decrease the prediction accuracy.

### Comparison of the Applications of Three Methods in Predicting ADMET Properties

**Figure 6:**
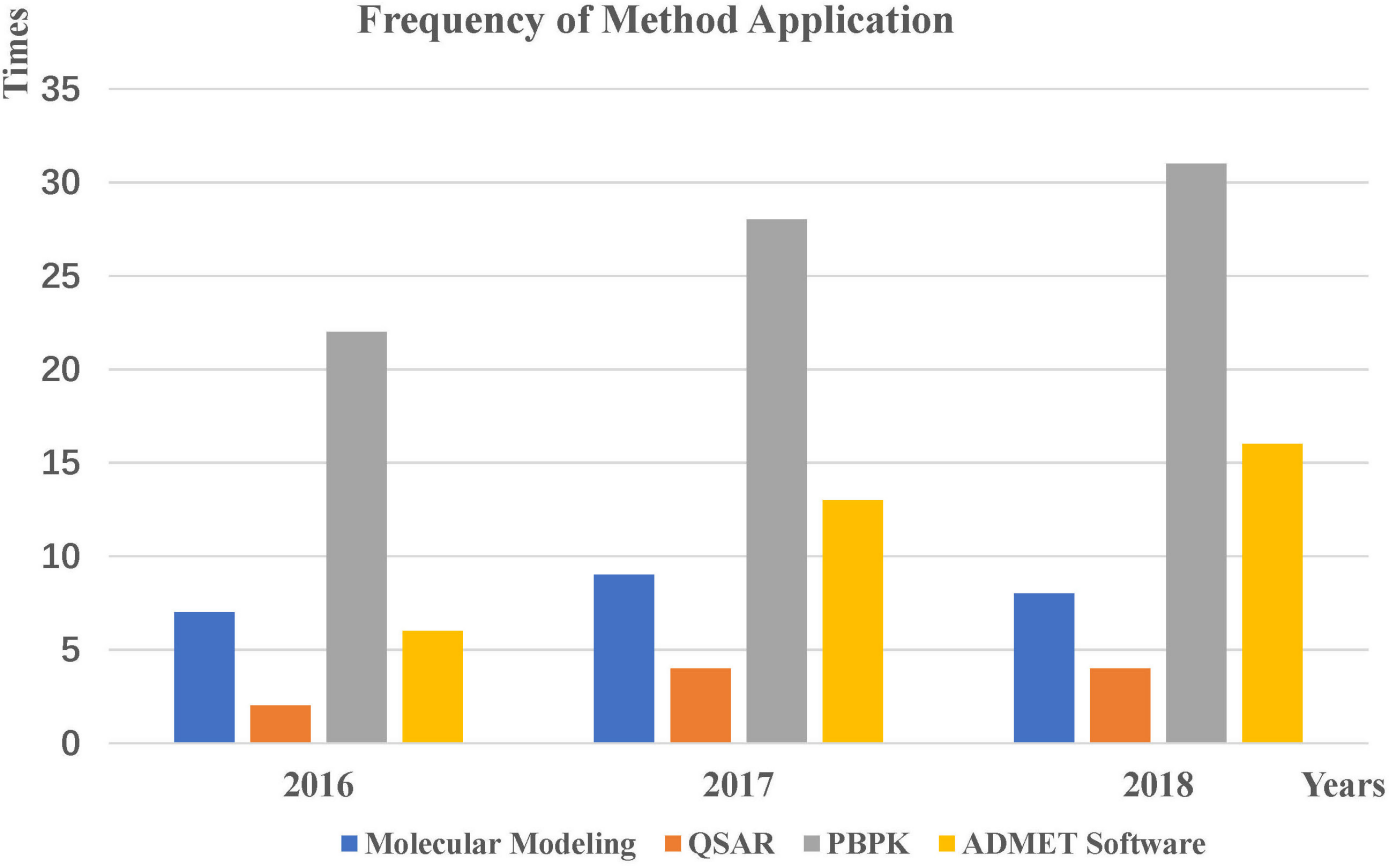
Application trends for each method from 2016 to 2018.

To compare the application trends of each method more intuitively, we counted the number of applications of these three methods from 2016 to 2018 (Figure 6). Applications using PBPK modeling software have exhibited an upward trend in recent years, which means that this approach will be the mainstream pharmacokinetic evaluation in the future. We believe that more user-friendly software will be developed, making the prediction process more convenient for users. Applications using ADMET software for forecasting are also on the rise. The main reason for these changes may be the demand for multi-property prediction and the phenomenon of drug recall, leading to the hope of predicting more pharmacokinetic properties in advance to reduce drug development costs. This increase also confirms that researchers have gradually integrated software predictions into the early stages of drug discovery to improve the success rate of drug development through complementarity and collaboration. In addition, we found that the QSAR method is rarely used alone, potentially due to the limitations of the QSAR method prediction. Therefore, we can increase or integrate the data volume of some specific QSAR models to generate a more comprehensive prediction model. Researchers can also combine different modeling methods and then perform relevant predictions. If a combined model can predict the properties well, then it can be used as a consensus approach to improve ADMET prediction accuracy.

### Previous Review and Prospective Studies of ADMET and PBPK Simulations

To date, there have been many reviews on ADMET and PBPK, which provide summaries from different perspectives. We found 11 articles that are closely related to this review after careful retrieval of the published literature. We have classified these articles into three categories based on the perspective of the descriptions: (1) directly related to our review, (2) machine learning (ML) methods, and (3) advances in *in silico* ADMET modeling.

Five reviews are directly related to this article. Yamashita and Hashida (2004) reviewed the application of structure-based methods and QSAR methods in predicting ADMET in detail. Cheng et al. (2013) introduced the recent progress and current challenges of QSAR in predicting ADMET and then discussed several new promising research directions that could be employed for systemic *in silico* ADMET prediction. Alqahtani (2017) reviewed the *in silico* models for predicting the ADMET properties of compounds and provided a comprehensive overview of the latest modeling methods and algorithms, as well as the application prospects of PBPK in predicting pharmacokinetics. Wang et al. (2015) briefly introduced the development of *in silico* models for ADMET prediction. They also focused on the modeling approaches, related applications, and potential advantages or disadvantages of these models used in drug discovery. Wishart (2007) highlighted ADMET property prediction, as well as ADME-related databases and software, and briefly introduced the application of PBPK and related software in ADMET.

Three out of 11 reviews are about machine learning methods for predicting ADMET. Ferreira and Andricopulo (2019) provided a detailed description of current machine learning approaches to ADMET modeling, focusing on key advances from 2017 to 2018. Tao et al. (2015) reviewed the progress of machine learning methods in ADMET prediction and discussed the performance, applications, and challenges in developing machine learning methods. Maltarollo et al. (2015) described the applications of ML methods in ADMET prediction.

The remaining three reviews addressed the progress of ADMET modeling. Specifically, Lin et al. (2003) introduced the desirable properties of new chemical entities (NCEs) from an ADMET perspective and discussed basic concepts, important tools, reagents, and experimental approaches used by researchers in predicting human pharmacokinetics. van de Waterbeemd and Gifford (2003) summarized the endpoints of pharmacokinetics, metabolism, and toxicity. Wang and Hou (2009) introduced the properties of ADMET, discussed the latest corresponding *in silico* models, and provided a brief summary of some software and databases. These articles introduced ADMET, PBPK, and related research progress from different perspectives.

## Conclusion

In this review, we provide a detailed and comprehensive introduction to currently used approaches or tools in predicting ADMET properties, including the basic principles, classification, and applications. In addition, we collect related applications from published articles over the past 3 years and analyze the trends in these applications. The purpose of this review is to help readers quickly understand these approaches and the characteristics of the related tools (databases and software). It may also provide readers with a better understanding of how existing tools can be applied to pharmacokinetic predictions. We are convinced that more accurate predictions due to users' familiarity with existing online services will increase the importance of *in silico* ADMET prediction in pharmacokinetics. In the future, we expect not only a reduced failure rate in drug development and drug recalls but also a faster timeline from R&D to market, as well as decreased costs during the late stage of development.

## Author Contributions

ZH, FW, and YZ contributed to the design and conception of the study. LL, YZ, FW, XS, GC, XW, XL, and MT performed information retrieval and analysis. FW, YZ, LL, and XS wrote the manuscript. YZ, FW, LL, XS, GC, XW, XL, and MT created the tables and figures. ZH and FW guided the manuscript writing and revised the manuscript. ZH provided financial support. All authors contributed to manuscript revision and have read and approved the submitted version.

## Conflict of Interest

The authors declare that the research was conducted in the absence of any commercial or financial relationships that could be construed as a potential conflict of interest.
